# Photobiomodulation Therapy in the Management of Orofacial Neuropathic Pain—WALT Position Paper 2026

**DOI:** 10.3390/jcm15031304

**Published:** 2026-02-06

**Authors:** Reem Hanna, Roberta Chow, Snehal Dalvi, Praveen R Arany, René-Jean Bensadoun, Alan Roger Santos-Silva, Jan Tunér, James D Carroll, Michael R Hamblin, Juanita Anders, Shimon Rochkind, Vladimir Heiskanen, Judith E. Raber-Durlacher, E-Liisa Laakso

**Affiliations:** 1 Department of Restorative Dental Sciences, Eastman Dental Institute, Medical Faculty, University College London, London WC1E 6DE, UK; 2 Head and Neck Academic Centre, Integrated Research, Division of Surgery and Interventional Science, Medical Faculty, University College London, London W1W 7TY, UK; 3 Dental Faculty, Royal College of Surgeons Ireland, 21-122 St Stephen’s Green, Dublin 2, D02 H903 Dublin, Ireland; 4 Faculty of Medicine and Health, University of Sydney, NSW, Brain and Mind Centre, 94 Mallett Street, Camperdown, NSW 2050, Australia; roberta.chow@sydney.edu.au; 5 Department of Periodontology, Swargiya Dadasaheb Kalmegh Smruti Dental College and Hospital, Nagpur 440001, India; dr.dalvisnehal@gmail.com; 6 Department of Oral Biology, School of Dental Medicine, University of Buffalo, Buffalo, NY 14215, USA; prarany@buffalo.edu; 7 Centre de Haute Énergie, 10 Bd Pasteur, 06000 Nice, France; rjbensad@gmail.com; 8 Oral Diagnosis Department, Piracicaba Dental School, State University of Campinas, 901 Limeira Avenue, Bairro Areão, Piracicaba, São Paolo P.O. Box 52, Brazil; alan@unicamp.br; 9 Private Dental Practice, Sollentuna, 19162 Stockholm, Sweden; tuner.jan@gmail.com; 10 Thor Photomedicine, Units 1, Anglo Office Park, White Lion Rd., Amersham HP7 9FB, UK; james.carroll@thorlaser.com; 11 Laser Research Centre, Faculty of Health Sciences, University of Johannesburg, Johannesburg 2092, South Africa; hamblin.lab@gmail.com; 12 Department of Anatomy, Physiology and Genetics, Uniformed University of the Health Sciences, 4301 Jones Bridge Road, Bethesda, MD 20814, USA; juanita.anders@usuhs.edu; 13 Department of Neurosurgery, Peripheral Nerve Reconstruction Division, Faculty of Medicine, Tel-Aviv University, 6 Weizmann Street, Tel Aviv 6423906, Israel; rochkind@zahav.net.il; 14 Microsurgical Center for Peripheral Nerve Reconstruction, Assuta Medical Center, 20 HaBarzel Street, Ramat HaHayal, Tel Aviv 6971020, Israel; 15 Private Dental Practice, Spider Med Hammasklinikka Manse, 3300 Tampere, Finland; valtsu.heiskanen@gmail.com; 16 Department of Oral Medicine, Academic Centre for Dentistry Amsterdam (ACTA), University of Amsterdam and VU University, 1081 Amsterdam, The Netherlands; judith@raber.nl; 17 Department of Oral and Maxillofacial Surgery, Amsterdam UMC, University of Amsterdam, 1105 Amsterdam, The Netherlands; 18 Mater Research Institute, University of Queensland, Level 2 Aubigny Place, Raymond Terrace, South Brisbane, QLD 4101, Australia; liisa.laakso@mater.uq.edu.au

**Keywords:** glossopharyngeal neuralgia, idiopathic trigeminal neuralgia, level of evidence, neuropathic pain, occipital neuralgia, PBM, photobiomodulation, post-herpetic neuralgia, post-traumatic trigeminal neuralgia, primary burning mouth syndrome

## Abstract

**Disclaimer:**

This position paper is based on the recommendations from the *15th WALT Congress (PBM2024)*, held in London from 23 to 25 August 2024, and is further informed by a follow-up review of current evidence and the clinical observations of an international, multidisciplinary panel of clinicians and researchers with expertise in neuropathic pain in orofacial conditions and/or the clinical application and dosimetry of photobiomodulation (PBM). This article is intended for use by healthcare professionals and researchers, and for information purposes only. As with all clinical guidelines, content should be interpreted in the context of ongoing research and evolving clinical practice, which may lead to new insights and recommendations over time. The views expressed reflect the consensus of the contributing panel at the time of publication and do not necessarily represent those of individual authors. The authors assume no responsibility for clinical decisions or actions taken based on the content presented herein.

**Abstract:**

**Background/Objectives**: Photobiomodulation (PBM) therapy has shown potential in managing orofacial neuropathic pain (ONP); however, inconsistent PBM dosimetry and methodological variability limit its clinical application. This World Association for Photobiomodulation Therapy (WALT) Position Paper aims to critically appraise current evidence and provide recommendations for Clinical Practice Guidelines (CPG) and Expert Consensus Opinion (ECO) where appropriate. **Methods**: Evidence evaluation was guided by the HANNA (Holistic Analysis & Novel Normative Actions) Framework, a structured multi-step methodology integrating systematic review, quality appraisal, and expert consensus. A systematic review was conducted in accordance with PRISMA 2020 guidelines. Methodological quality was assessed using validated tools: AMSTAR 2 for systematic reviews, RoB2 for randomized controlled trials (RCTs), and ROBINS-I for non-randomized studies (NRCTs). The AGREE II Reporting Checklist was applied to ensure transparency and rigor in the development of WALT recommendations. The Somerfield Criteria were used to rate the level of evidence (LoE) for each included ONP condition, where deemed appropriate. **Results**: WALT CPG were established for primary burning mouth syndrome (BMS), supported by robust evidence (LoE I) from 204 patients across six “Low RoB” RCTs and NRCTs, and 557 patients included in a “High-Confidence” systematic review and meta-analysis of “low RoB” RCTs. WALT ECO were developed for idiopathic trigeminal neuralgia (TN) and post-herpetic neuralgia (PHN), both supported by LoE II. Insufficient evidence precluded formal recommendations for post-traumatic trigeminal neuralgia, glossopharyngeal neuralgia, and occipital neuralgia. **Conclusions**: This Position Paper introduces the HANNA Framework, for the first time, as a robust and transparent methodology for developing WALT recommendations by delivering evidence-based CPG for PBM in the management of neuropathic pain associated with primary BMS, along with ECO for both TN and PHN. These recommendations support PBM as a safe and effective therapeutic approach, and provide a structured roadmap for future research and periodic guidelines updates.

## 1. Introduction

### 1.1. Background and Rationale

Neuropathic pain (NP) is a chronic and often debilitating condition arising from lesions or diseases that affect the somatosensory system, distinct from nociplastic pain, characterized by altered nociceptive processing in the absence of identifiable tissue or somatosensory system lesions [1]. It affects millions of individuals globally and is associated with significant impairments in quality of life (QoL), increased incidence of mood disorders, and functional disability [2]. Current first-line pharmacological treatments (such as tricyclic antidepressants, serotonin-norepinephrine reuptake inhibitors, gabapentinoids, and topical agents) have limitations [3,4] (see Section 1.4 below).

Photobiomodulation (PBM) therapy, formerly known as low-level laser therapy (LLLT), involves the use of lasers and light-emitting diodes (LEDs). It has gained attention as a non-invasive, non-thermal, non-pharmacological intervention for chronic pain conditions [5,6], including NP [7,8]. PBM utilizes red or near-infrared (NIR) light (typically within the 600–1100 nm wavelength range) to stimulate photochemical and photobiological processes at the cellular level [9]. The primary mechanism involves the absorption of photons by cytochrome c oxidase, a mitochondrial enzyme, which results in enhanced production of adenosine triphosphate (ATP) and activation of downstream pathways associated with anti-inflammatory, neuroprotective, and analgesic effects [10].

### 1.2. Mechanistic Basis of PBM in Neuropathic Pain

**Figure 1:**
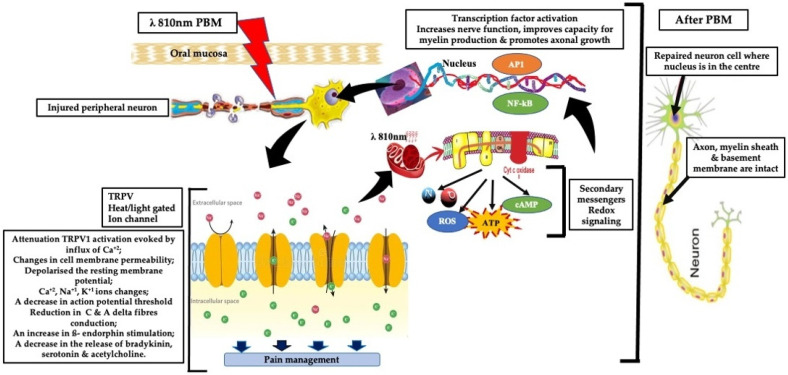
Schematic representation of the effects of photobiomodulation (PBM) on injured peripheral neurons through its primary, secondary, and tertiary mechanisms. PBM triggers signaling pathways that contribute to the attenuation of neuropathic pain and the downregulation of pro-inflammatory cytokines and neurotransmitter mediators [11] (Permission obtained from Hanna et al.). Abbreviations: PBM: photobiomodulation; PBMT: photobiomodulation therapy; TRPV1: transient receptor potential cation channel subfamily V member 1; Ca^+2^: calcium ion; K^+1^: potassium ion; Na^+1^: sodium ion; cyt c oxidase: cytochrome c oxidase; CAMP: cyclic adenosine monophosphate; ROS: reactive oxygen species; ATP: adenosine triphosphate; NO: nitric oxide; AP1: activation protein1; NF-kB: nuclear factor kappa.

The therapeutic relevance of PBM in NP is supported by several key mechanisms (Figure 1) [11]:

- Mitochondrial activation: PBM enhances mitochondrial respiration and ATP synthesis through cytochrome c oxidase activation, promoting cellular energy availability and repair processes [10,12].
- Anti-inflammatory effects: PBM has been shown to downregulate pro-inflammatory cytokines such as TNF-α, IL-1β, IL-6, and substance P, thereby mitigating neuroinflammation [8,13]
- Neuroprotection and regeneration: PBM upregulates neurotrophic factors like nerve growth factor (NGF) and supports axonal repair, which are crucial for peripheral nerve regeneration [14].
- Analgesic modulation: PBM modulates nociceptive signaling via effects on ion channels (e.g., TRPV1) and peripheral nerve excitability. Experimental animal and ex vivo studies show that, under specific irradiation conditions, PBM can transiently reduce action potential amplitudes and alter fast axonal transport in small-diameter A∂ and C fibers, leading to reduced nociceptive signaling propagation. These effects are neuromodulatory, parameter-dependent, and reversible, and at clinically applied, non-thermal doses, analgesia is more likely mediated by modulation of nerve function and inflammatory pathways rather than true conduction block [15,16,17].
- A third mechanism involving activation of extracellular latent TGF-β1 has been noted, which is primarily responsible for the tissue resilience, healing, and regeneration [18].
- Central modulation and thermoregulation: Functional imaging studies suggest PBM may influence central pain processing and reduce neurogenic inflammation [19].

These mechanisms align closely with the pathophysiology of NP, supporting PBM’s potential as a therapeutic modality across various NP syndromes, particularly those with mixed peripheral and central involvement.

### 1.3. Relevance to Orofacial Neuropathic Pain

Orofacial neuropathies such as primary burning mouth syndrome (BMS), idiopathic trigeminal neuralgia (TN), post-herpetic neuralgia (PHN), post-traumatic trigeminal neuropathy (PTTN), glossopharyngeal neuralgia (GPN), and occipital neuralgia (ON) are often peripheral in origin but may evolve to involve central sensitization [20,21,22]. The following briefly summaries the predominant peripheral and central contributions in each condition:

- Primary BMS:
- TN is primarily peripheral at onset, usually due to neurovascular compression, with central sensitization developing in chronic cases.
- PHN begins with peripheral nerve injury, which often shows early central involvement.
- PTTN is mainly peripheral, with a central mechanism emerging in persistent pain.
- GPN and ON are predominantly peripheral, though central sensitization can occur in prolong cases.

Clarifying the peripheral and central contributions in each condition is important for guiding targeted interventions. PBM, which modulates both peripheral and central mechanisms, may therefore serve as a valuable adjunctive therapy in this context.

### 1.4. Limitations of Current Treatments for Neuropathic Pain

Despite the widespread use of pharmacological interventions for NP, their effectiveness and limitations vary considerably depending on the underlying disease entity (e.g., idiopathic TN, PHN) [3,4]. Overall, several key limitations remain clinically relevant across many, though not all, NP conditions:

- Incomplete or inconsistent pain relief: Pharmacological treatments for NP often provide only partial analgesia, with substantial inter-individual variability. While certain conditions (e.g., TN) may initially respond well to specific agents, sustained pain control is often difficult to maintain over time, and long-term efficacy remains limited for a substantial proportion of patients.
- Adverse effect profiles: Many first-line agents are associated with dose-limiting adverse effects, including sedation, dizziness, cognitive impairment, and dry mouth, which can compromise tolerability, adherence, and overall QoL. The clinical impact of these effects varies across NP subtypes and patient populations.
- Risk of tolerance, dependence, and misuse: Opioid therapies, in particular, pose risks of tolerance development, physical dependence, and misuse, limiting their suitability for long-term management of chronic NP and raising important safety concerns.
- Lack of disease-modifying or regenerative effects: Current pharmacological treatments primarily provide symptomatic relief and do not promote neuronal repair or address underlying pathophysiological mechanisms, thereby failing to alter disease progression across NP conditions.
- Variable efficacy in conditions involving central sensitization: In NP states characterized by central sensitization, conventional pharmacotherapies often demonstrate reduced effectiveness, as they inadequately target the central neural mechanisms sustaining chronic pain.

These shortcomings underscore the need for integrative treatment approaches that not only alleviate symptoms but also promote underlying tissue repair and neuroregeneration, goals aligned with PBM’s proposed mechanisms.

### 1.5. Aims and Objectives

This position paper aims to evaluate the current evidence on PBM therapy in the management of orofacial NP, focusing on primary BMS, idiopathic TN, PHN, PTTN, GPN, and ON.

The objectives include:

- To systematically review and synthesize the available evidence supporting PBM use in orofacial neuropathies.
- To critically appraise the clinical effectiveness of PBM in managing orofacial neuropathic pain (ONP).
- To establish the level of evidence (LoE) for PBM effectiveness in the included ONP conditions.
- To identify gaps in current research and propose directions for standardization and future investigations.
- To formulate WALT recommendations based on Clinical Practice Guidelines and Expert Consensus Opinions, explicitly reflecting the strength and quality of the underlying evidence.

## 2. Evidence Evaluation and HANNA Framework

**Figure 2:**
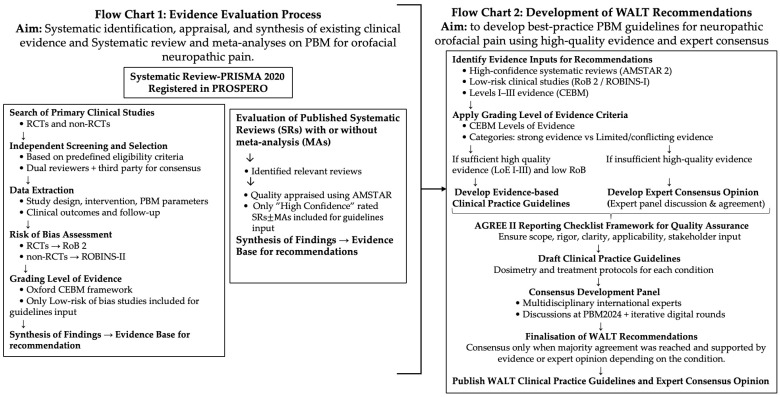
Overview of the HANNA (Holistic Analysis & Novel Normative Actions) Framework used to develop WALT recommendations. Flow Chart 1 (**left panel**): Structured process of evidence evaluation. Flow Chart 2 (**right panel**): Structured process used to develop WALT recommendations (clinical practice guidelines and expert consensus opinion) derived from Flow Chart 1.

This position paper was developed using a structured, evidence-based, multi-step methodology that integrated systematic literature evaluation, quality assessment, and expert consensus. The methodology is referred to as the HANNA (Holistic Analysis & Novel Normative Actions) Framework. The process described in the manuscript (Figure 2) represents the original work of the first author and is reported here for the first time.

The HANNA Framework provides a comprehensive and robust stepwise workflow integrating evidence identification, appraisal, synthesis, and expert consensus for the development of WALT Recommendations. This framework structured the development of the position paper, ensuring that all recommendations, clinical practice guidelines, and expert opinions are based on a rigorous appraisal of the available evidence.

### 2.1. Evidence Evaluation and Quality Assessment

As illustrated in Flow Chart 1–Figure 2, the HANNA Framework guided the systematic identification, appraisal, and synthesis of available evidence on PBM for OFN, with the systematic review performed in accordance with PRISMA 2020 guidelines [23] and including randomized controlled trials (RCTs), non-randomized clinical trials (NRCTs), and existing published systematic reviews (SRs), with or without meta-analyses (MAs). This dual-layered approach, while potentially introducing overlapping data, was intentionally selected to allow triangulation and validation of findings without inflating evidence strength. Two authors independently conducted the review, and any discrepancies or disagreements were resolved through consultation with a third author. To minimize the risk of overlap and double-counting, all the included studies were carefully cross-checked, and duplicates were excluded during data synthesis.

The level of evidence (LoE) for PBM in each orofacial neuropathic pain condition, including primary BMS, idiopathic TN, PHN, PTTN, GPN, and ON, was determined, where possible, using the adapted Somerfield Criteria [24] (Table 1). This assessment was based primarily on clinical studies with low risk of bias, supplemented, when possible, by SRs with or without MAs rated “High Confidence”.

To ensure the inclusion of well-designed, evidence-based studies suitable for translation into World Association for Photobiomodulation Therapy (WALT) Guidelines or Expert Consensus Opinions, a rigorous qualitative analysis was performed. The included studies were assigned Level I, II, or III evidence based on the Oxford Centre for Evidence-Based Medicine (CEBM) criteria [25]. CEBM Levels IV–VII were not used for evidence inclusion or grading.

Within this framework, the methodological quality and risk of bias of the included studies were assessed using validated assessment tools. The included SRs, with or without MAs, were appraised using A Measurement Tool to Assess Systematic Reviews 2 (AMSTAR 2) [26]. RCTs were evaluated using the Cochrane *Risk of Bias 2* (RoB 2) tool [27], and NRCTs using the Risk of Bias in Non-Randomized Studies of Interventions (ROBINS-I) tool [28]. All the assessments were conducted independently by two authors, with discrepancies resolved through discussion or consultation with a third author.

Finally, proposed WALT Recommendations, including both Expert Consensus Opinions and Clinical Practice Guidelines, were evaluated using the AGREE II Reporting Checklist [29] to ensure transparency, methodological soundness, and applicability of guideline development and reporting process. This integrated approach ensures that evidence evaluation, quality assessment, and recommendation development are systematic, rigorous, and reproducible.

### 2.2. Scientific Rationale and Novelty of the HANNA Framework

The HANNA Framework adapts established methodological standards for systematic review, risk of bias assessment, quality appraisal, and assignment of level of evidence, while applying AGREE II to ensure transparency in recommendations. Its novel contribution lies in unifying these tools into a structured, multi-step workflow that incorporates dual-layered triangulation of primary studies and systematic reviews, along with the stepwise development of WALT Recommendations based on both Expert Consensus Opinions and Clinical Practice Guidelines. Reported here for the first time, the HANNA Framework ensures reproducibility, transparency, and methodological rigor beyond that of any individual existing framework.

## 3. Materials and Methods

### 3.1. Evaluation of the Existing Evidence for Systematic Review

#### 3.1.1. Primary Clinical Studies

We conducted a systematic search and evaluation of current RCTs and NRCTs using predefined eligibility criteria aligned with the scope of this position paper. The systematic review for this position paper was prospectively registered with PROSPERO (International Prospective Register of Systematic Reviews) (ID: CRD42020198921) and was conducted in accordance with the PRISMA 2020 Guidelines [23].

#### 3.1.2. Search Strategy

The search strategy included only terms related to or describing the study domain and intervention, conducted by two review authors (R.H., S.D.) independently. The latter also screened the studies independently, and a template of the relevant data was produced. To assess inter-reviewer reliability analysis, a kappa (κ) statistic ≥ 0.8 was deemed acceptable [30]. In case of any inconsistencies in ratings, a third review author (V.H.) was consulted to reach consensus.

Searches were conducted in April 2025 across multiple databases using both Subject Headings (MeSH) terms and relevant keywords in MEDLINE (PubMed, PMC), EMBASE, CINAHL, Cochrane Library, Scopus, ClinicalTrials.gov, and Cochrane Central Register of Controlled Trials (CENTRAL), ScienceDirect, and Google Scholar.

Topic-specific journals were hand searched including: Photobiomodulation, Photomedicine and Laser Surgery (and its predecessor Photomedicine and Laser Surgery), Clinical Oral Investigation, Journal of Dental Research, Lasers in Medical Science, Journal of Photochemistry and Photobiology, Photodiagnosis and Photodynamic Therapy, Scientific Report, Photodermatology Photoimmunology & Photomedicine, Journal of Biophotonics, Indian Journal of Dermatology, Venereology and Leprology, Dental Research Dental Clinics Dental Prospects, Journal of Clinical Medicine, The Clinical Journal of Pain, Antioxidants, Pain Journal, Journal of Orofacial Pain, and Laser Therapy. The reference lists of all included studies were assessed to obtain additional eligible papers. The search strategy included only terms relating to or describing the study domain and intervention. The terms were combined with the Cochrane MEDLINE filter for controlled trials of interventions. An attempt to obtain unpublished studies was undertaken by a formal screening through https://ClinicalTrials.gov (Accessed on 18 April 2025) using the same keywords of the search strategy. Grey literature sources were screened by searching relevant databases and institutional websites using a manual review of titles and summaries.

To obtain a thorough, sensitive, and specific approach for the electronic search, the use of relevant free text keywords as well as MeSH terms that were logically connected with the help of appropriate Boolean operators was performed. The Boolean operators “**AND**” and “**OR**” were used to improve the search strategy through various combinations. The following terms were searched in combination:

“Photobiomodulation” **OR** “PBM” **OR** “low-level laser therapy” **OR** “LLLT”

**AND**

“Orofacial neuropathic pain” **OR** “neuropathic orofacial pain” **OR** “trigeminal neuralgia” **OR** “idiopathic trigeminal neuralgia” **OR** “primary burning mouth syndrome” **OR** “post-traumatic trigeminal neuropathy” **OR** “trigeminal nerve injury” **OR** “BMS” **OR** “PHN” **OR** “post-herpetic neuralgia”, **OR** “glossopharyngeal neuralgia” **OR** “neuropathic pain” **OR** “occipital neuralgia” **OR** “Burning sensation” OR “BMS” **OR** “GPN” **OR** “ON” **OR** “PTTN” **OR** “TN”.

#### 3.1.3. Data Extraction

From each eligible study, we extracted: study design and condition treated, sample size and intervention groups, PBM parameters (wavelength, power, irradiance, fluence, spot size, irradiation area and exposure time), treatment protocols (frequency, duration, number of sessions, application method), clinical outcomes (pain scores, functional recovery, QoL measures, statistical significance), follow-up duration, adverse effects and compliance.

#### 3.1.4. Eligibility Criteria

Inclusion Criteria

- Human RCTs and NRCTs evaluating PBM therapy in patients diagnosed with NP in the following orofacial conditions: primary BMS, idiopathic TN, PHN, PTTN, GPN, and ON.
- Studies reporting pain outcomes, functional recovery, or QoL metrics.
- Articles published in English.
- Studies reporting PBM parameters and treatment protocols.
- Systematic reviews and meta-analyses on PBM evaluation in the management of NP.
- Peer-reviewed papers published up to March 2025.

Exclusion Criteria

- *In vitro* and *in vivo* animal studies.
- Case series, retrospective, case reports, or short communications.
- Studies focused on NP induced by neurodegenerative conditions and oncology treatments.
- Studies investigating PBM for trigeminal nerve (V) regeneration or neurosensory recovery, rather than for NP.
- Any other orofacial conditions-induced pain and unrelated to NP.
- Studies involving combined cohorts of various orofacial pain conditions.
- Studies without a clear methodology or intervention details.
- Studies focused exclusively on non-NP.
- Studies focused on tumor or infection-induced NP.

#### 3.1.5. Risk of Bias Assessment

To assess methodological rigor and minimize bias, and to assess the quality of studies investigating PBM for ONP, we applied two validated tools. The Cochrane Risk of Bias 2 (RoB 2) tool for RCTs was used to assess risk across five domains, rating each study as low, some concerns, or high risk of bias. ROBINS-I for NRCTs was used to evaluate bias across seven domains, resulting in overall judgments ranging from low to critical risk. These assessments were tailored to the study designs available for each condition. Where both RCTs and NRCTs were present, both tools were used to ensure comprehensive evaluation.

All assessments were conducted independently by two reviewers. Inter-rater agreement was measured using Cohen’s kappa (κ = 0.92), and disagreements were resolved through discussion or third-party adjudication.

Only studies with low risk of bias were considered for inclusion in the development of WALT recommendations.

#### 3.1.6. Level of Evidence and Grading Framework

The Oxford CEBM Levels of Evidence system was used to rank the strength and validity of the evidence. This hierarchical framework distinguishes the quality of evidence based on study design, methodological rigor, and risk of bias. It is widely used in evidence-based medicine to guide clinical decisions and research. Table 2 outlines the CEBM criteria. The included studies in the systematic review (PRISMA 2020) comprised RCTs, NRCTs, and published SRs ± MAs of RCTs, which were assigned Levels I, II, and III, respectively, based on CEBM [25]. Only studies meeting these evidence levels were considered for inclusion and grading.

### 3.2. Evaluation of the Current Published Systematic Reviews

Systematic reviews and meta-analyses addressing PBM for neuropathic orofacial pain were identified and critically appraised using AMSTAR 2 (A Measurement Tool to Assess Systematic Reviews 2) to evaluate the methodological quality of each review. AMSTAR 2 does not produce an overall score but instead identifies critical and non-critical weaknesses (Supplementary File S1).

It comprises 16 items: seven critical and nine non-critical. Reviews were classified into four levels of confidence (high, moderate, low, or critically low) depending on the number and severity of methodological weaknesses. Critical domains include protocol registration, comprehensive literature search, justification for exclusions, risk of bias assessment, appropriateness of meta-analyses, consideration of bias in interpretation, and assessment of publication bias. Non-critical domains include duplicate study selection, data extraction, and reporting funding sources.

Two authors (V.H. and L.L.) independently evaluated the included systematic reviews. To minimize potential bias, all assessments strictly followed AMSTAR 2 criteria and were independently reviewed by a third assessor (S.V.D.) with no involvement in the authorship of either the reviews or this paper.

### 3.3. Development of WALT Guidelines and Recommendations

The development of the WALT of guidelines and recommendations was guided by a structured and transparent process that integrated the best available evidence with expert consensus, following universally recognized appraisal tools.

#### 3.3.1. Consensus and Grading Process

Recommendations and guidelines were primarily informed by:

- High confidence of existing SRs ± MAs of RCTs appraised using the AMSTAR 2 tool (LoE I according to CEBM);
- Systematic review (PRISMA 2020) of low-risk-of-bias clinical studies, RCTs, and NRCTs, assessed using RoB 2 and ROBINS-I, respectively, and graded as Level II or III evidence, respectively, according to the Oxford CEBM–LoE framework.

Where high-quality evidence was insufficient, recommendations were developed through “expert consensus opinion”. In such cases, consensus was reached only when multiple studies with consistent findings were available, and a majority agreement was achieved among the expert panel.

The overall objective of this process was to extrapolate the most reliable PBM dosimetry and treatment protocols for each condition, forming the evidence base for updated WALT recommendations.

After initial search and evaluation of the evidence, the AGREE II instrument was applied as a quality assurance checklist to ensure that the development process of the guideline meets internationally recognized standards in terms of scope, rigor, stakeholder involvement, and applicability (Figure 2).

#### 3.3.2. AGREE II Checklist Adaptation

The AGREE II (Appraisal of Guidelines for Research and Evaluation) Reporting Checklist was adapted for this study and used by the authors (Supplementary File S2) to guide the development and reporting of WALT’s recommendations and to enhance their trustworthiness, reproducibility, and clinical implementation. This framework ensured quality and transparency by evaluating: Scope and purpose; Stakeholder involvement; Rigor of development; Clarity of PBM protocol and dosimetry; Applicability; and Editorial independence. For this position paper, the results of the AGREE II appraisals are provided in the Supplementary File S3.

Three authors independently completed the AGREE II assessments and subsequently held virtual meetings to discuss the outcomes and resolve any disagreements by consensus. To further enhance objectivity, an external reviewer also reviewed the assessments and assisted in resolving any remaining discrepancies. This approach ensured a rigorous and transparent evaluation supporting the development of the WALT recommendations.

### 3.4. Consensus Development Panel

The WALT consensus recommendation statements were developed through a structured, iterative process involving a multidisciplinary, international panel of experts in neurology, pain medicine, PBM, oral medicine, rehabilitation, oncology, neurorehabilitation, and evidence-based practice. Collectively, the panel brought over 200 years of clinical and research experience in PBM, NP, and orofacial NP.

Initially, the expert panel met and discussed the proposed development of the paper during the PBM2024-WALT conference in London, August 2024. The consensus process then included multiple discussion rounds in person and digitally, critical appraisal of evidence, and final agreement on recommendations for each condition. Data from the curated literature were analyzed for specific parameters and careful analysis for reported and calculated parameters to generate consensus using the recently described dosimetry guidelines [31].

## 4. Results

This section presents the synthesized findings from key sources of evidence, including selected RCTs and NRCTs studies, and relevant systematic reviews, and derives recommendations for clinical practice guidelines and expert consensus opinions to guide research for orofacial neuropathies. For three specific conditions from the list of neuropathies explored, various amounts of published evidence were available for review, and each has been described separately.

Although there is some overlap between the clinical studies included in this systematic review and those cited in previous systematic reviews, the present position paper distinguishes itself by employing a rigorous and methodologically robust approach that warrants a re-examination of the available evidence. This approach includes the use of stricter inclusion criteria, a comprehensive appraisal of both RCTs, NRCTs, and existing reviews, and the development of targeted clinical recommendations based solely on methodologically sound evidence. This integrated and critically filtered synthesis provides a more reliable and clinically actionable understanding of the role of PBM in managing ONP.

### 4.1. Summary Assessment of All Included Studies and Published Reviews

**Figure 3:**
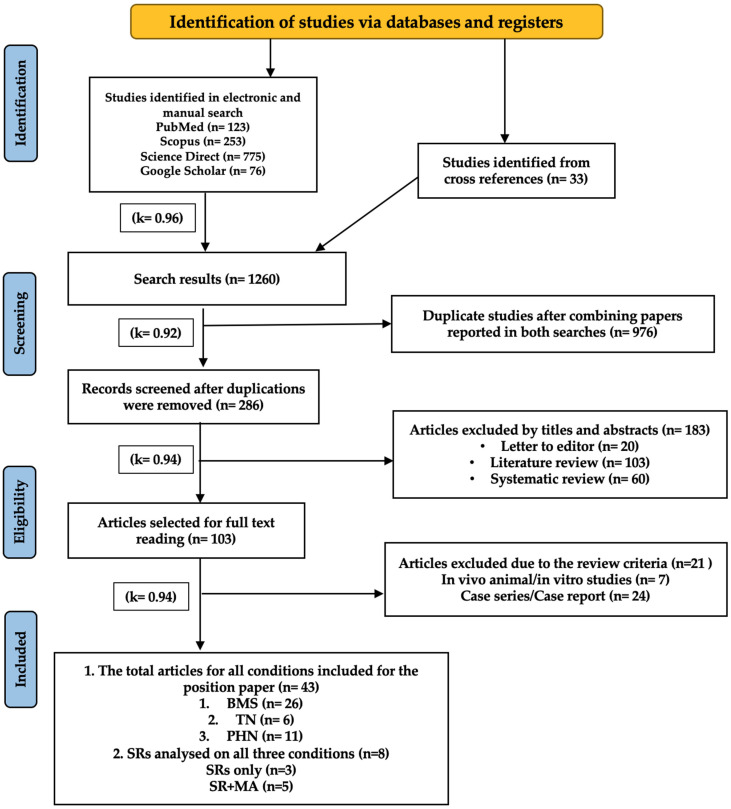
PRISMA 2020 Flow Diagram showing the studies included in our systematic review, as well as the existing published systematic reviews ± meta-analyses identified and included in the chart.

Figure 3 presents the PRISMA flow diagram [23], illustrating the search strategy and selection process of included clinical studies and existing published SRs and MAs, based on the predefined eligible criteria. The review followed the PRISMA 2020 framework and was registered with PROSPERO (CRD42020198921).

A total of eight SRs ± MAs was included in the final assessment: three SRs [32,33,34] and five SRs and MAs [35,36,37,38,39] (κ = 0.94). Of these, only one, conducted by Hanna et al. 2021 [35] for BMS, was rated as “High Confidence” and informed the WALT recommendations, indicating methodology quality and reliable conclusions. Three reviews [36,37,38] were rated as “Moderate Confidence” (more than one non-critical weakness but still useful), and four reviews [32,33,34,39] were rated critically “Low Confidence” (multiple critical flaws, making conclusions unreliable). Two authors independently evaluated all included systematic reviews, including the “High Confidence” SR, and to minimize potential bias, all assessments strictly followed AMSTAR 2 criteria and were independently reviewed by a third assessor with no involvement in the authorship of any included reviews (κ = 0.92). These results are presented in Figure S1 in Supplementary File S7.

Among the six orofacial neuropathic conditions evaluated in this review, BMS was the only condition supported by a High Confidence SR and MA; in addition, primary clinical studies for BMS were also included in the present review. For all other conditions, evidence synthesis relied exclusively on primary clinical studies.

In total, 43 clinical studies met the inclusion criteria: primary BMS: 26 studies [11,40,41,42,43,44,45,46,47,48,49,50,51,52,53,54,55,56,57,58,59,60,61,62,63,64]; idiopathic TN: 6 studies [65,66,67,68,69,70]; PHTN: 11 studies [71,72,73,74,75,76,77,78,79,80,81] (κ = 0.94). No eligible clinical studies were found for GPN, ON, or PTTN.

Details of the excluded studies, along with reasons for exclusion, are provided in Supplementary File S4. The PRISMA 2020 checklist is available in Supplementary File S5.

Information on the included studies in the present systematic review, including the LoE for each, is presented in Supplementary File S6. The risk of bias (RoB2) for each included study and the AMSTAR 2 critical level of confidence rating for the included published SRs and MAs are outlined in Supplementary File S7 (Figures S1–S10).

### 4.2. Assessment of Evidence from Systematic Review and Meta-Analysis in BMS

The effectiveness and dosimetry of PBM in primary BMS were evaluated based on the “High Confidence” SR and MA by Hanna et al. 2021 [35] (LoE I, according to CEBM criteria), which synthesized data from RCTs considered LoE II (according to CEBM) for each trial. The meta-analysis included 236 patients across four RCTs and demonstrated a statistically significant reduction in neuropathic pain based on visual analog scale (VAS) scores (MD = −1.47, 95% CI: −2.40 to −0.53; *p* = 0.002). Additional outcomes, including anxiety and QoL, were evaluated in 321 patients across five RCTs. Despite variability in PBM parameters, Hanna et al. 2021 [35] proposed a protocol based on higher-quality trials.

WALT recommendation for PBM therapy in generalized orofacial neuropathies align with the protocol proposed by Hanna et al. 2021 [35] and include the following parameters: near-infrared wavelength; power output of 200–300 mW measured with a power meter; continuous wave (CW) emission mode; energy per point of ~6 J; irradiation time of 30–60 seconds (s) per point, and two sessions per week over five consecutive weeks (total of 10 sessions).

The pooled evidence of this “High Confidence” SR and MA supports the effectiveness and recommended dosimetry of PBM in the management of NP in BMS. These findings are reflected in the detailed synthesis of the included primary RCTs and NRCTs (low RoB) presented in Section 4.3, which forms the basis of the WALT Clinical Practice Guidelines.

### 4.3. Assessment of Evidence of Included Studies for Each Orofacial Neuropathic Condition

#### 4.3.1. Primary Burning Mouth Syndrome

All 26 included studies [11,40,41,42,43,44,45,46,47,48,49,50,51,52,53,54,55,56,57,58,59,60,61,62,63,64] underwent a rigorous RoB assessment, of which only six were rated as having a low risk of bias (Figures S2–S5 in Supplementary File S7), with a total of 204 patients recruited across these six studies.

As this position paper aimed to prioritize studies with low RoB, only these six studies were used to inform the recommendations. These included four RCTs [40,41,42,43] classified as LoE II (Supplementary File S6) and two NRCTs [11,57] classified as LoE III (Supplementary File S6) according to OCEBM criteria. The six studies were evaluated to inform evidence-based PBM dosimetry, treatment protocols, and suggested outcome measures for potential inclusion in clinical recommendations (Supplementary File S6). The evaluated studies [11,40,41,42,43,57] are as follows:

De Pedro et al. 2020 [40]: This single-blind RCT evaluated the effect of PBM versus (vs.) sham controlled in patients with BMS, using an 810 nm diode laser in pulsed mode. The treatment was delivered at a power output of 600 mW in CW, with an irradiance of 1.2 W/cm^2^, an energy of 6 J per point, and a fluence of 12 J/cm^2^, across 56 standardized oral mucosal sites. The study demonstrated sustained reductions in NP and improvements in psychological well-being.

Bardellini et al. 2019 [41]: This double-blind RCT evaluated the efficacy of PBM compared to placebo. The PBM protocol employed simultaneous multiwavelength emission at 660, 800, and 970 nm, operating at 50% duty cycle. The device delivered an average power output of 3200 mW, applied in a continuous moving probe over the 150 cm^2^ affected area. Treatment consists of 11 phases (21 s per phase), for a total irradiation time of 231 s per session. The average irradiance was 3.2 W/cm^2^, based on a 1 cm^2^ beam spot size. The protocol resulted in a total energy delivery of 739.2 J per session. Participants received weekly treatment for 10 weeks, which led to a significant reduction in NP and improvement in oral health-related QoL (OHIP-14).

Sugaya et al. 2016 [42]: This RCT evaluated the effectiveness of PBM vs. placebo in patients with BMS. A 790 nm laser in CW delivered at 120 mW, 6 J/cm^2^, and an irradiance of 4 W/cm^2^, over 4 sessions across 2 weeks. Outcomes were similar between the PBM and placebo groups, with minimal short-term differences, suggesting a possible psychogenic component to symptomatology.

Arduino et al. 2016 [43]: This RCT evaluated the efficacy of PBM vs. pharmacotherapy (topical clonazepam) in patients with BMS. A 980 nm laser was delivered at 300 mW in a CW, with a fluence of 10 J/cm^2^, administered twice weekly over five weeks. PBM demonstrated superior improvements in NP (VAS, MPQ) and QoL (OHIP-49) at 8–12 weeks of follow-up.

Hanna et al. 2022 [11]: This prospective comparative feasibility trial evaluated the efficacy of PBM vs. pharmacotherapy in patients with oral NP in primary BMS based on a long-term follow-up. PBM was applied using an 810 nm diode laser at therapeutic power out of 200 mW (utilized power) measured with a power meter, 6 J/cm^2^, 30 s per point, 9 intra-oral irradiating points, including the affected trigeminal nerve-lingual nerve and trigger areas (painful points), with an irradiance of 1.97 W/cm^2^, delivered twice weekly over five consecutive weeks. PBM demonstrated a rapid and sustained reduction in NP (VAS 7.6 → 3.9) and improvement in functionality and overall well-being over a 9-month follow-up, which were statistically significant compared to pharmacotherapy, with no reported adverse effects.

De Abreu et al. 2024 [57]: This single-arm prospective feasibility trial evaluated the long-term effects of PBM therapy on NP and health-related QoL in patients with BMS. Treatment involved biweekly sessions × 12 months, using a 660 nm laser at 100 mW, 60 s per point, delivering 6 J per point. The laser was applied directly to the affected oral areas following a centimetric grid pattern to ensure uniform coverage. Over 12 months, participants demonstrated statistically significant improvements in EQ-5D–5L scores.

Table 3 presents the dosimetry and treatment parameters reported in the six included studies, with original values shown in non-italic text and the calculated values derived from the reported data presented in italic text. These findings formed the basis for the WALT Clinical Practice Guidelines, supporting the effectiveness of PBM in primary BMS. Reinforcing this evidence base, the included published systematic review and meta-analysis [35] classified as Level I evidence according to OCEBM criteria and rated as “High Confidence” using AMSTAR, evaluated 236 patients across four RCTs and demonstrated a statistically significant reduction in oral NP intensity in primary BMS (MD = −1.47; 95% CI: −2.40 to −0.53; *p* = 0.002). Secondary analyses, including 321 patients from five RCTs, also reported improvements in anxiety and QoL (Section 4.2).

Taken together, these high-quality, low-risk-of-bias studies, along with the Level I, “High-Confidence” meta-analysis, provide robust support for the WALT-recommended PBM parameters and justify their adoption in the Clinical Practice Guidelines. This evidence confirms the clinical efficacy and reproducibility of PBM therapy in the management of NP in primary BMS.

**WALT Recommendation: Clinical Practice Guidelines**

The strength of the current evidence is based on “Low RoB” RCTs and NRCTs [11,40,41,42,43,57], as well as a “High-Confidence” SR and MA [35], demonstrating the clinical benefit of PBM in the management of NP in patients with primary BMS.

Table 4 summarizes the WALT recommendations for PBM dosimetry and treatment protocols for managing NP in BMS. These recommendations were derived from six included Low RoB studies [11,40,41,42,43,57] (Table 3), along with the findings of nine RCTs reported in the High-Confidence SR and MA [35] (Section 4.2). Collectively, this evidence base provides a strong foundation for the WALT clinical practice guideline recommendations and for informing future RCTs.

It is important to note that employing a multiwavelength protocol (red and NIR) [43] allows for safe and uniform energy distribution over a large area while maintaining the benefits of higher instantaneous irradiance at the probe tip for effective tissue penetration. This is particularly advantageous when the clinician needs to cover a large target surface area with a moving probe, thereby reducing chairside treatment time.

Regarding safety and precautions, PBM therapy demonstrates a safety profile across all included studies and published SR and MA, with no reported adverse effects. Validated patient-reported outcomes measures (PROMs) for evaluating NP in patients with primary BMS include: pain intensity (0–10 VAS); pain quality (McGill Pain Questionnaire, MPQ); oral function and oral health-related QoL (OHIP-14 or OHIP-49); general health status (EQ-5D-5L); and psychological well-being (anxiety and depression scales).

Based on the current evidence, the LoE for PBM in this condition, according to the Somerfield Criteria, is rated as Level I (Strong Evidence). The completed AGREE II checklist, which supports WALT recommendations—clinical practice guidelines—in using PBM as an effective and safe intervention for this condition, is provided in Supplementary File S3.

#### 4.3.2. Idiopathic Trigeminal Neuralgia

Out of the six included studies [65,66,67,68,69,70], only three RCTs [67,69,70] with a low risk of bias (RoB) (Figures S6 and S7 in Supplementary File S7) were prioritized for this position paper, which aimed to emphasize clinical studies with a low RoB. A total of 98 patients were recruited across these three studies.

These three RCTs (LoE II) studies investigated the use of PBM in TN and were used to inform the clinical practice guidelines or expert consensus. The studies were assessed for methodological quality, PBM parameter reporting, outcome measures, and follow-up (Supplementary File S6) to determine their suitability for inclusion in a WALT clinical recommendation.

Ebrahimi et al. 2018 [67] conducted an RCT comparing carbamazepine alone to carbamazepine combined with adjunctive PBM therapy for the treatment of TN. The intervention group received nine PBM sessions over three weeks (three sessions per week), resulting in a significantly greater reduction in NP, as measured by the VAS, compared to the control group receiving carbamazepine alone (*p* = 0.003). PBM was applied to identified trigger points or, if absent, to 2–3 sites along the patient’s reported pain pathway extraorally (V1–V3 at the skin). Treatment utilized 810 nm in CW mode, delivering 5 J per point over 25 s at a maximum output power of 200 mW, suggesting deeper photonic penetration. The probe tip had a 1 cm diameter. They followed up with the patient at 1 month and ensured pain alleviation sustainability.

Eckerdal et al. 1996 [69] conducted a double-blind, placebo-controlled trial administering weekly PBM over five weeks. At one-year follow-up, six of 16 patients in the treatment group remained pain-free, compared to only one of 14 in the placebo group. The report also noted reduced analgesic use in the treatment group. The essential PBM parameters were reported, and the remaining parameters were calculated based on the provided data.

Walker et al. 1987 [70] employed a 632.5 nm laser. The emission mode was pulsed at a 50% duty cycle and 20 Hz, delivering an average power output of 0.477 mW with a power density of 47.6 mW/cm^2^. The spot size was 4 mm^2^ (0.04 cm^2^ at the skin).

Three painful peripheral facial areas (V1–V3) were irradiated following a progressively increasing exposure schedule: 30 s (Week 1), 45 s (Week 2), 60 s (Weeks 3–6), and 90 s (Weeks 7–10). The use of progressively longer exposure times reflects Walker’s clinical observation that irradiating the painful area for a full minute at the outset of therapy commonly exacerbated pain. This exacerbation was not observed when laser exposure was introduced gradually over successive treatments. Walker et al. 1987 [70] did not attribute this effect to tissue heating. Similar observations have been reported by others, with some patients experiencing transient symptom aggravation before subsequent improvement following PBM. 

Despite using a 632 nm wavelength and a lower power output delivered in pulsed emission mode, Walker’s stepped-dose strategy resulted in a significant reduction (*p* < 0.002) in NP intensity and frequency across the treatment period. This study also provided some of the most detailed PBM parameters available in the literature, enhancing reproducibility.

Table 5 summarizes the reported dosimetry and treatment protocols of these studies, (presented in non-italic text), and the interpreted data derived from them (presented in italic text). Based on these data, WALT formulated the recommendations outlined below based on expert consensus opinion.

**WALT Recommendation: Expert Consensus Opinion**

PBM demonstrated clinically meaningful reductions in TN pain intensity and frequency across all three included studies, with no adverse effects reported, supporting a favorable safety and tolerability profile. PBM, when used along with pharmacological therapy, has been associated with improved NP outcomes and may help optimize overall pain management in TN by reducing medication dependence in selected patients [67]. However, current evidence in TN is insufficient to support PBM as a complete substitute for pharmacological treatment.

Table 6 summarizes WALT recommendations for idiopathic TN, which are based on expert consensus opinion informed by the included studies assessed as having “Low RoB” (Table 5).

A progressive increase in irradiation parameters is suggested to optimize patient tolerability. For extra-oral irradiation, consideration of the optical properties of target tissues is essential to ensure optimal energy delivery to the intended surface area. The use of a power meter to accurately measure the therapeutic power output reaching the target surface area, as well as thermography to monitor the cutaneous temperature, is strongly recommended in clinical studies.

Standardization of PBM dosimetry and treatment protocols, including wavelength, light source, emission mode, power density reaching the target surface area, fluence, energy, and irradiation time per point, treatment duration, and treatment frequency, is critical to improve reproducibility and facilitate clinical implementation. PBM irradiation should be directed toward pain trigger points and the nerves involved in the pathology, following their anatomical distribution. Comprehensive documentation of all PBM dosimetry parameters is essential to ensure reproducibility and support standardized clinical application.

Pain outcomes in the included studies were predominantly assessed using VAS, with limited use of multidimensional or functional pain assessments. These methodological limitations highlight the need for well-designed, adequately powered RCTs employing standardized PBM dosimetry, comprehensive outcome measures, including QoL and functional outcomes, and long-term follow-up.

Based on the available clinical studies, the LoE for PBM in idiopathic TN was classified as Level II according to the adapted Somerfield Criteria, reflecting moderate strength for this condition. The AGREE II Reporting Checklist was applied to assess the methodological quality and transparency of the expert consensus opinion underlying the WALT recommendations. The completed checklist is provided in Supplementary File S3 and does not influence the assigned LoE.

#### 4.3.3. Post-Herpetic Neuralgia

As this position paper aimed to prioritize clinical studies with “Low RoB”, only three of the eleven included clinical studies [71,72,73,74,75,76,77,78,79,80,81], specifically studies [74,77,81] with low RoB (Figures S8–S10 in Supplementary File S7), were used to inform the clinical practice guidelines or expert consensus opinion.

These included two RCTs (double-blind, crossover, placebo-controlled) [74,81] and one NRCT [77], all of which evaluated PBM for herpes zoster-related neuropathic pain, with a primary focus on PHN (Supplementary File S6). A total of 103 patients were recruited across these three studies.

Interestingly, only one of the RCTs [72] was excluded for “Serious RoB” (Figure S10 in Supplementary File S7); this excluded study investigated acute herpes zoster ophthalmicus (HZO) using 830 nm LED-PBM therapy.

The details of the included studies in the position paper are outlined below:

Toshikazu et al. 1997 [74] applied an 830 nm laser PBM near the stellate ganglion at two power settings (60 mW and 150 mW) for 3 min in 8 PHN patients, targeting a single point at the stellate ganglion anterior to C7/T1. They observed that 150 mW is more effective than 60 mW. There was a clear dose-dependent reduction in pain on VAS, along a significant increase in regional skin temperature assessed by thermography after PBM treatment, with no such corresponding effects in the placebo condition. These physiological changes were interpreted as sympathetic modulation, supporting the hypothesis that PBM near the stellate ganglion may produce effects similar to a stellate ganglion block.

Kemmostsu et al. 1991 [77] conducted a double-blind, non-randomized crossover trial involving 63 PHN patients, applying an 830 nm laser to multiple points across the painful dermatome and near the stellate ganglion. Pain was measured using a 0–10 numerical rating scale. In the immediate phase, 26 patients experienced “very good” pain relief (score 0–3), and 30 experienced “good” relief (score 4–7) after the first session. Over a mean of 36 treatment sessions, 12 patients achieved complete pain relief, and 46 had residual but reduced pain. Temperature was monitored. Significant increases in skin surface temperature and reductions in pain were observed compared to placebo, although a mild placebo effect was also noted. No adverse events were reported.

Moore et al. 1988–1989 [81] studied 17 PHN patients using an 830 nm diode laser in a randomized crossover design. The exact irradiation or trigger points were not specified, but treatment was applied over the painful areas, likely involving multiple points along the affected dermatome, without targeting the stellate ganglion. The authors reported statistically significant improvements in both pain intensity and pain distribution following active PBM treatment compared to placebo, based on repeated assessments after each of eight sessions (four active, four sham). These findings provide further support for the short-term analgesic effects of PBM in PHN.

All three studies used 830 nm GaAlAs diode lasers, not LEDs, and collectively support short-term analgesic effects of PBM in PHN, though the targeted anatomical sites, treatment protocols, and outcome measures varied. Additionally, long-term efficacy remains unproven, and multidimensional outcome assessments were lacking. These studies, although conducted between 1988 and 1997, continue to provide relevant data on PBM dosimetry and treatment protocols, which may support the design of future clinical trials in harnessing PBM in the management of PHN.

Table 7 summarizes the reported dosimetry and treatment protocols of these studies (presented in non-italic text), and the calculated and interpreted data (presented in italic text). Based on these data, WALT formulated the recommendations outlined below based on expert consensus opinion.

**WALT Recommendation: Expert Consensus Opinion**

PBM at 830 nm with an output power of 60 mW has consistently produced clinically meaningful reductions in NP intensity and distribution in patients with PHN [74,77,81]. However, a higher power of 180 mW appears to be more effective for reaching deeper anatomical targets, such as the stellate ganglion.

PBM irradiation sites varied across the studies: a single point on the stellate ganglion [74], whereas others targeted ~7–12 points along the branches of the trigeminal nerve (V1–V3), depending on the affected branch or any combination of branches [77,81]. Notably, deep-seated tissues like the stellate ganglion require longer exposure times (180 s) with applied pressure [74], whereas more superficial tissues, such as the trigeminal nerve branches, respond to shorter durations of 10–15 s [77,81].

In terms of treatment frequency, 2–3 sessions per week are advisable. The total treatment duration depends on the onset and chronicity of the condition as well as the patient’s response, up to 36 sessions in total.

Outcome evaluation has primarily relied on unidimensional tools such as VAS and pain mapping, without integrating functional or multidimensional pain assessments. The absence of long-term data limits conclusions regarding the durability of PBM therapeutic effects, highlighting the need for long-term follow-up.

It should be noted that the included studies were conducted between 1988 and 1997. While diagnostic standards and clinical trial methodologies have evolved, these findings provide valuable preliminary insights and support the promise of PBM in PHN management. Contemporary, well-designed RCTs are warranted to confirm effectiveness, durability, and clinical applicability.

Expert consensus considers PBM a safe and potentially effective adjunctive therapy, particularly in refractory PHN cases. Table 8 presents WALT recommendations for PHN, reflecting expert consensus opinion based on the studies assessed as having “Low RoB” (Table 7). The adapted AGREE II Reporting Checklist was applied to ensure methodological quality and transparency (Supplementary File S3).

Based on the adapted Somerfield Criteria, the LoE for PBM in PHN is Level II, reflecting moderate-quality evidence from well-designed RCTs and NRCTs. This classification aligns with the approach used for idiopathic TN and indicates that, while the findings are promising, further larger, standardized RCTs with multidimensional outcomes and long-term follow are warranted to confirm both effectiveness and durability of PBM, and to determine whether maintenance dosing is needed.

#### 4.3.4. Post-Traumatic Trigeminal Neuralgia (PTTN)

No clinical studies were identified that evaluated the effects of PBM on NP induced by PTTN as a distinct cohort. Consequently, it is not possible to provide WALT recommendations, clinical practice guidelines, or expert consensus opinion for this condition, or LoE based on Somerfield Criteria.

We encourage the development of well-designed clinical studies specifically targeting PTTN, guided by preliminary PBM dosimetry protocols derived from pilot studies and emerging evidence, and long-term follow-up to support future consideration in WALT recommendations. 

#### 4.3.5. Glossopharyngeal (GPN) and Occipital Neuralgia (ON)

To date, no studies have investigated the application of PBM in GPN and ON, and theretofore, no WALT recommendations (clinical practice guidelines or expert consensus opinion) can be made for these conditions. We also cannot rate the LoE based on Somerfield for these conditions.

We advocate for the design of high-quality clinical studies focused specifically on these conditions. Future studies should be informed by emerging evidence and preliminary PBM dosimetry protocols established in pilot research, and should include long-term follow-up to strengthen the case for future inclusion in WALT recommendations.

## 5. Path Forward for Advancing PBM Research in Orofacial Neuropathic Pain

Clinical guidelines represent one of the most effective tools to support healthcare professionals in translating the current evidence base into daily practice. The World Association for Photobiomodulation Therapy (WALT) recognized the need to establish evidence-based PBM dosimetry and treatment protocol recommendations, beginning with orofacial NP conditions as the first in a planned series.

As outlined in this position paper, the WALT recommendations, comprising both clinical practice guidelines and expert consensus opinion statements, will undergo periodic review, with comprehensive revisions planned every three years to maintain alignment with new evidence and evolving clinical best practices in the management of orofacial NP.

This position paper is endorsed by multiple international professional associations in the field of PBM, underscoring a growing consensus on the role of PBM therapy in managing orofacial NP [Australian Medical Photobiomodulation Association (AMPA), Asia Pacific Laser Institute (APLI), European Medical Laser Association (EMLA), Academy of Laser Dentistry (ALD) (see *Acknowledgments*)]. These endorsements strengthen the credibility of the recommendations and support the clinical relevance and translational potential of PBM in this context.

Over the past decade, significant advances in basic science have laid a strong foundation for the clinical application of PBM in orofacial NP. To further support its integration into practice, ongoing research should build on emerging evidence indicating that PBM therapy may serve as an effective adjunct to transcutaneous electrical nerve stimulation or pharmacotherapy [49,82].

The guideline development process highlighted recurring methodological considerations in the literature, such as dosimetry reporting, calibration of delivered energy, patient cohort characterization, and targeted anatomical application. Addressing these considerations provides a roadmap to strengthen study design, optimize PBM protocols, and enhance clinical outcomes. Based on this, the following research and reporting priorities are proposed to advance PBM evidence and clinical implementation in ONP:

- **Standardized and Complete Dosimetry Reporting**

All relevant PBM parameters and treatment characteristics should be comprehensively reported to ensure experimental reproducibility and enable meaningful comparisons across studies [83]. Essential PBM parameters include wavelength, output power (therapeutic—measured with a power meter), emission mode, energy per point, irradiance, energy density, and spot size/beam area. Equally important, treatment specifications must also be fully reported, including irradiation time per point, irradiation points (number, location, and sites), treatment frequency and total duration, laser–tissue distance, treated area (s) (local, distal, or both), and irradiation technique (spot, scanning, or continuous sweeping motion). When applicable, the grid pattern or sequential application method should also be described.

- **Verification of Delivered Dose**

The use of calibrated power meters should be standard practice in both research and clinical settings to verify the therapeutic power output delivered to the target tissue, ensuring accurate and effective dosing, and thereby optimizing clinical outcomes [84,85]. In addition, further optical and radiometric characterization should be performed at the device output to ensure optimal therapeutic performance. This includes measuring the beam area using the D4σ second-moment method with a beam profiler, determining irradiance with the same profiler, and assessing peak wavelength and spectral bandwidth using a spectrometer [86].

- **Choice of Light Source**

Lasers and LEDs operating at the same wavelength, with identical irradiance and beam area, should theoretically produce similar biological effects [87,88].

Devices, whether laser or LED, with tips designed for firm contact with the skin and emitting in the NIR range at powers in the hundreds of milliwatts, can penetrate tissue to depths of up to 4 cm, depending on tissue type, beam geometry, and application condition [89]. It is certainly possible for therapeutically effective power to be achieved at depths of 4 cm, provided surface power densities are used that do not overheat the overlying skin. This depth of penetration allows potential modulation of deeper structures, such as the stellate ganglion, which is relevant for managing orofacial NP. Preclinical data suggest that PBM can modulate dorsal root ganglion neurons and reduce nociception, indicating neuromodulatory effects at the ganglion or nerve level [90].

While lasers are often marketed as superior due to coherence and polarization, photobiological evidence indicates that the wavelength and delivered photon dose are the primary determinants of tissue penetration and physiological effects, rather than the coherence or type of light source [87,88].

- **Condition-Specific Protocols**

PBM parameters should be tailored to the distinct pathophysiological mechanisms of each orofacial NP condition, rather than applying generalized treatment protocols.

- **Pathophysiology-Informed Application**

Protocol development should incorporate current understanding of disease mechanisms and PBM’s biological effects to inform dosing strategies and treatment planning [11].

- **Anatomical Precision in Treatment Delivery**

PBM irradiation should be guided by detailed clinical and diagnostic evaluation, with precise anatomical targeting of affected neural structures, their distributions, and trigger symptomatic points [11]. This includes both extra-oral (cutaneous) and intra-oral sites, selected according to the patient’s specific symptom patterns and underlying pathophysiology.

- **Thermographic Monitoring During PBM**

It is recommended to implement infrared thermography, a non-contact imaging device, to monitor cutaneous temperature in real time [91], and this can be useful during PBM application. Thermography provides continuous visual feedback of tissue temperature during irradiation. The target temperature should remain below 45 °C [92] to maintain non-thermal and safe treatment conditions. This monitoring is particularly important in PBM applications using an extra-oral approach for managing NP induced by TN and PHN, where maintaining thermal safety is essential to prevent discomfort or exacerbation of pain.

- **Improved Study Design**

Future clinical trials should adopt rigorous methodological standards, including clearly defined inclusion and exclusion criteria, appropriate randomization, control groups, and blinded outcome assessments, to minimize bias and improve reliability. The importance of considering long-term follow-up is crucial to determine the duration of PBM effectiveness and whether a maintenance dose is required. In addition, the device’s optical characteristics should be verified by suitably trained optical personnel before and after the trial to ensure proper performance. Further guidance on these and radiometric measurements is provided in “Verification of Delivered Dose” above.

WALT recognizes the importance of addressing these methodological, technical, and clinical challenges. These suggestions lay the groundwork for developing condition-specific PBM guidelines and reinforce WALT’s commitment to promoting the safe, effective, and evidence-based use of PBM in the management of orofacial NP. Through its modulation of mitochondrial function, inflammation, and nerve regeneration, PBM targets several key mechanisms underlying NP while avoiding the systemic side effects commonly associated with pharmacological treatments.

The growing body of clinical evidence, along with PBM’s favorable safety and tolerability profile [11,64,93], supports its potential as a promising therapeutic option in the management of oral NP. The National Institute for Health and Care Excellence (NICE) is currently evaluating the role of PBM in the management of oral neuropathic pain. The topic is under consideration for further development, pending additional evidence and broader awareness. This review is linked to NICE Topic ID: GID-IPG10373 [94], based on a report submitted by the first author of this position paper (ID number 1547), aiming to contribute to the ongoing assessment.

## 6. Conclusions

For the first time, this paper introduces the novel and robust HANNA (Holistic Analysis & Novel Normative Actions) Framework, a structured, evidence-based methodology integrating systematic literature evaluation (PRISMA 2020), quality appraisal, evidence synthesis, and expert consensus. The framework employs validated assessment tools to ensure that the resulting evidence synthesis, ensuring that the resulting WALT recommendations are rigorous, transparent, and evidence-driven.

Applying this framework, WALT Clinical Practice Guidelines were established for primary BMS, while Expert Consensus Opinions were developed for idiopathic TN and PHN, reflecting differences in evidence strength. PBM is supported as a safe and effective therapeutic approach, with clinically applicable dosimetry parameters identified for primary BMS, idiopathic TN, and PHN. Formal recommendations could not be made for PTTN, GPN, and ON, due to insufficient evidence.

The paper provides a structured roadmap and recommendations to advance PBM research in ONP, promoting standardized protocols and studies with direct clinical relevance. This roadmap identifies key focus areas to strengthen study design, optimize PBM dosimetry, enhance clinical outcomes, and improve evidence quality.

Regular updates are planned every three years to incorporate emerging evidence and maintain the relevance of the WALT recommendations.

## Figures and Tables

**Table 1:** Somerfield Criteria [24].

Level	Definition
I	Evidence from at least one properly designed randomized controlled trial (RCT) or meta-analysis of RCTs.
II	Evidence from well-designed non-randomized controlled trials, cohort studies, or case-control studies.
III	Evidence based on expert opinion, clinical experience, descriptive studies, or case reports.
IV	Evidence from multiple time series or dramatic results in uncontrolled experiments.
V	Evidence from Expert committee reports, clinical experience, or descriptive studies.

**Table 2:** Oxford Centre for Evidence-Based Medicine Levels of Evidence [25].

Level	Description
I	Evidence from a systematic review of all relevant randomized controlled trials (RCT’s), or evidence-based clinical practice guidelines based on systematic reviews of RCT’s
II	Evidence obtained from at least one well-designed randomized controlled trial (RCT)
III	Evidence obtained from well-designed controlled trials without randomization, quasi-experimental
IV	Evidence from well-designed case-control and cohort studies
V	Evidence from systematic reviews of descriptive and qualitative studies
VI	Evidence from a single descriptive or qualitative study
VII	Evidence from the opinion of authorities and/or reports of expert committees

**Table 3:** Summary of the PBM dosimetry and treatment protocols from the included studies on primary BMS which determined the WALT Recommendations, summarized in Table 4. Parameters reported directly in the included studies are presented in non-italic text. Italicized values in indicate calculated estimates derived from the interpretation of the reported parameters and the device programs used. These estimates were used to calculate photon energy, photon fluence, and Einstein values.

First Author, Year, and Citation	PBM Dosimetry and Treatment Protocols													
	λ (nm) Light Source	Emission Mode	Power (mW)	Irradiance (W/cm^2^)	Energy (J)/ Point	Irradiation Points (Number and Location)	Fluence (J/cm^2^)/ Point	Beam Area (cm^2^)	Irradiation Time (s)/ Point	Frequency per Week/Rx Duration	Follow-Up	Photon Energy (eV)	Photon Fluence (*p*.J/cm^2^)	Einstein (ɇ)
Hanna et al. 2022 [11]	810 (laser)	CW	200	1.97	6	9–Tongue (tip, dorsum, ventral) and along V3 and lingual nerves (Spot technique)	6 (Total 59.1)	0.088	30	2× a week/5 consecutive weeks	Up to 9 months	1.5	*88.656*	*19.7*
de Pedro et al. 2020 [40]	810 (laser)	CW	600	1.2	6	56–Lip, tongue, vestibular and buccal mucosa, hard palate (spot technique)	12	0.5	10	2× a week/5 consecutive weeks	>4 months	1.5	*18*	*4*
Bardellini et al. 2019 [41]	660, 800, 970 (laser)	Pulsed/ 50% duty cycle, 1–20,000 Hz	3200	3.2	*4.93*	Continuous sweeping movement *over 150 cm^2^* treatment areas: tongue (lateral, dorsum, tip), lips, buccal mucosa	*4.93*	150 (1 cm^2^ continuous sweeping motion)	Total 231	1× a week/ 10 weeks Rx	>1 month	1.9 1.3	*1404.5* *960.9* *2365.4*	*525.6*
Sugaya et al. 2016 [42]	790 (laser)	CW	120	4	1	Tongue, palate, lips, buccal mucosa in centimetric grid pattern	6	0.03	50	2× a week/2 consecutive weeks	7,14,30,60 and 90-day	1.5	*9*	*2*
Arduino et al. 2016 [43]	980 (laser)	CW	300	1	3	Depending on the painful areas: lip, tongue, buccal mucosa, palate (spot technique)	10	0.28	10	2× a week/ 5 weeks	8–12 weeks	1.3	*13*	*2.9*
de Abreu et al. 2024 [57]	660 (laser)	CW	100	*0.1*	*6*	The tongue, buccal mucosa, and hard palate are irradiated in centimetric grid pattern	6	1	60	2× a week/ 5 weeks	12 months	1.9	*11.4*	*2.5*

**Table 4:** WALT Recommendation 2026—Clinical Practice Guidelines for NP Management in Patients with Primary BMS (LoE I, according to Somerfield Criteria—Strong Recommendation).

	PBM Dosimetry and Treatment Protocols														
λ (nm) Laser	Emission Mode	Power (mW)	Irradiance (W/cm^2^)	Energy (J)	No. of Irradiation Points/Areas	Irradiation Location	Irradiation Technique	Fluence (J/cm^2^)/ Point	Beam Area (cm^2^)	Irradiation Time (s)	Frequency per Week/ Rx Duration	Laser- Tissue Distance	Photon Energy (eV)	Photon Fluence (*p*.J/cm^2^)	Einstein (ɇ)
		100–600		1–6/ point	9–52 points		Spot		0.03–1	10–60/ point		<1 mm in contact	1.3–1.9	*6.5–22.8*	*1.4–5.1*
660, 790–980 (Single or * Multi-wavelength, red and NIR)	CW/ * Pulsed		0.1–4 (* higher irradiances potentially thermal)			Intra and extra-oral of the affected areas, including V2 and V3		5–12			2× a week/5 consecutive weeks (Adjust to patient’s response)					
		* 3200 (* Average)		* 739 J per area *÷* 150 cm^2^ = 4.93 J/cm^2^	1 × 150 cm^2^ (Sum of all the treated areas)		* Con-tinuous sweeping motion (CSM)		150 (beam area 1 cm^2^ in CSM)	* Total 231		* 1.5 cm	1.3 and 1.9	*6.409* *9.367* *15.78*	*3.5*

* **Means potentially thermal depending on the treatment technique**. Please use a set of parameters from only one of the studies—AVOID mixing parameters from different studies. This table summaries observed parameter ranges across individual studies and is intended to describe the breadth of PBM device parameters for which clinical benefits have been achieved. In the source literature, the wavelength, power, irradiance, spot size, treatment time, anatomical target, and technique are tightly conserved within each individual protocol. Clinical application should therefore adopt a complete parameter set from a single study, and the parameters listed are not interchangeable.

**Table 5:** Summary of the PBM dosimetry and treatment protocols from the included studies on idiopathic trigeminal neuralgia (TN) which determined the WALT Recommendations, summarized in Table 6. Parameters reported directly in the included studies are presented in non-italic text. Italicized values indicate calculated estimates derived from the interpretation of the reported parameters and the device specifications used. These estimates were used to calculate photon energy, photon fluence, and Einstein values. Abbreviations: V: trigeminal nerve; V1: ophthalmic neve; V2: maxillary nerve; V3: mandibular nerve.

First Author, Year, and Citation	PBM Dosimetry and Treatment Protocols													
	λ (nm) Laser	Emission Mode	Power (mW)	Irradiance (W/cm^2^)	Energy (J)/Point	Irradiation Points (Number and Location)	Fluence (J/cm^2^)	Spot Size (cm^2^)	Irradiation Time (s)	No. Sessions/ Rx Duration	Follow-Up	Photon Energy (eV)	Photon Fluence (*p*.J/cm^2^)	Einstein (ɇ)
Ebrahimi et al. 2018 [67]	810	CW	200	*~0.2544*	5	2–3 cutaneous sites along the pain pathway of affected branches of V	6.36	*~* *0.79*	25	3× a week/ 3 weeks (total 9 sessions)	1 month	1.5	*7.5*	*1.6*
Eckerdal et al. 1996 [69]	832	CW	31	*~* *0.143*	2	1–5 facial painful area along the affected branches of V	9.2	*0.22*	*64.5*	1× a week/ 5 weeks	12 months	1.5	*4.8*	*1.1*
Walker et al. 1987 [70]	632.5	Pulsed, 20 Hz, 50% duty cycle	0.477 (Average)	0.0476	*0.0143*	Along V1–V3 (cutaneous)	*~* *0.36*	0.04 at skin	30—week 1	3× a week/ 10 weeks (Total 30 sessions)	Pain intensity assessed during Rx duration	1.9	*0.7*	*0.1*
					*0.0215*		*~* *0.54*		45—week 2				*1.0*	*0.2*
					*0.0286*		*~* *0.72*		60—week 3–6				*1.4*	*0.3*
					*0.0429*		*~* *1.07*		90—week 7–10				*0.7*	*0.1*
													*3.76*	*0.84*

**Table 6:** WALT Recommendation 2026—Expert Consensus Opinion for NP in Patients with Idiopathic TN (Level of Evidence II, according to Somerfield Criteria).

PBM Dosimetry and Treatment Protocols													
λ (nm) Laser	Emission Mode	Power (mW)	Irradiance (W/cm^2^)	Energy (J)/Point	Irradiation Points (Number and Location)	Fluence J/cm^2^	Spot Size (cm^2^)	Irradiation Time (s)/ Point	Frequency per Week/ Rx Duration	Laser– Tissue Distance	Photon Energy (eV)	Photon Fluence (*p*.J/cm^2^)	Einstein (ɇ)
810–832 (Single wavelength)	CW	31–200	~0.15–0.25	~2–5		~6.3–9					1.5	*9.4–13.5*	*2.1–3*
					6 cutaneous points along the affected V1-V3 branches (2 points per branch). V2 and V3 are the most commonly affected nerves, and involvement is usually unilateral		~0.04–0.79	25–90	1–3× a week/ 3–5 weeks, (Up to 10 weeks, depending on the patient’s response.	At skin, with applied of pressure			
632.5	Pulsed, 20 Hz, 50% duty cycle	0.477 (Average)	0.0476	0.014–0.043 (Stepwise increase)		~0.36⟶1.07 (Progressive)					1.9	*0.68–2.0*	*0.1–0.4*

**Please use a set of parameters from only one of the studies**—AVOID mixing parameters from different studies. This table summaries observed parameter ranges across individual studies and is intended to describe the breadth of PBM device parameters for which clinical benefits have been achieved. In the source literature, the wavelength, power, irradiance, spot size, treatment time, anatomical target, and technique are tightly conserved within each individual protocol. Clinical application should therefore adopt a complete parameter set from a single study, and the parameters listed are not interchangeable.

**Table 7:** Summary of the PBM dosimetry and treatment protocols from the included studies on PHN which determined the WALT Recommendations, summarized in Table 8. Parameters reported directly in the included studies are presented in non-italic text. Italicized values indicate calculated estimates derived from the interpretation of the reported parameters and the device specifications used. These estimates used to calculate photon energy, photon fluence, and Einstein values.

First Author, Year and Citation	PBM Dosimetry and Treatment Protocols													
	λ (nm)	Emission Mode	Power (mW)	Irradiance (W/cm^2^)	Energy (J)/Point	Irradiation Points	Fluence (J/cm^2^)	Spot Size (cm^2^)	Irradiation Time (s)/ Point	No. Sessions/ Rx Duration	Follow-Up	Photon Energy (eV)	Photon Fluence (*p*.J/cm^2^)	Einstein (ɇ)
Toshikazu et al. 1997 [74]	830	CW	60 (MLD-1002)	*0.48*	*10.8*	Single point—stellate ganglion (C7/T1) with applied pressure. Spot treatment technique	85.9	0.126 (4 mm)	180	2× a week (1× for 60 mW and 1× for 150 mW) -then crossover	Only 30 min post-Rx	1.5	*2063.7* *5159.2*	*458.6* *1146.5*
			150 (MLD-1003)	*1.19*	*27*		214.8		180					
Kemmostsu et al. 1991 [77]	830	CW	60 (MLD-2001)	~1.2–3	*~* *0.6*	V dermatome, tender, painful points (superficial points). Spot treatment technique *~7–12 points per clinical and reported parameters*	*30*	*0.02* *(0.6 W ÷ 3 W/cm^2^)*	10	2–3× a week at out-patients; 4–6× a week at in-patients/ 36 on average	Refers to average Rx duration 36 *± 12* (no post-Rx)	1.5	*5159.2* *10,318.5*	*1146.5* *2292.9*
Moore et al. 1988 [81]	830	CW	60	3	*0.9*	V dermatome (spot treatment technique). * ~10 points per clinical and* *reported parameters.*	45	*0.02*	15	2× a week/ 8 treatments in total	1 month	1.5	*2160*	*480*

**Table 8:** WALT Recommendation 2026—Expert Consensus Opinion for NP in Patients with PHN (Level of Evidence II, according to Somerfield Criteria).

PBM Dosimetry and Treatment Protocols													
λ (nm) Laser	Emission Mode	Power (mW)	Irradiance (W/cm^2^)	Energy (J)/Point	Irradiation Points (Number and Location)	Fluence (J/cm^2^)	Spot Size (cm^2^)	Irradiation Time (s)/Point	Frequency per Week/ Rx Duration	Laser- Tissue Distance	Photon Energy (eV)	Photon Fluence (*p*.J/cm^2^)	Einstein ( ɇ )
830	CW	60–150	0.48–3	~0.6–27 depending on irradiation time	7–12 points along V1-V3: Any combination of V1, V2, and V3 depends on where the shingles outbreak occurred: forehead: 2–3; around eye: 2–3; upper eyelid: 1–2; bridge of nose: 1–2; optional temple or lateral forehead: 1–2. 60 mW is effective for superficial points. One point to the stellate ganglion (C7/T1), whereby 150 mW is more effective than 60 mW	*30*–45 to the V branches (superficial); 85.9–214.8 to the stellate ganglion block	0.02–0.1257	10–15 to each point of V1-V3 branches; 180 for 1 point to the stellate ganglion block. Spot treatment technique	2–4× per week up to 36 sessions, adjusted per patient’s response	At skin (cutaneous contact) with applied pressure for deep-seated tissues	1.5	*173.85* *–389.7*	*38.63–86.6*

**Please use a set of parameters from only one of the studies.** AVOID mixing parameters from different studies. This table summaries observed parameter ranges across individual studies and is intended to describe the breadth of PBM device parameters for which clinical benefits have been achieved. In the source literature, the wavelength, power, irradiance, spot size, treatment time, anatomical target, and technique are tightly conserved within each individual protocol. Clinical application should therefore adopt a complete parameter set from a single study, and the parameters listed are not interchangeable.

## Data Availability

All the data are available in the text and Supplementary Files.

## Supplementary Materials

The following supporting information can be downloaded at: https://www.mdpi.com/article/10.3390/jcm15031304/s1, Supplementary File S1: AMSTAR 2 tool; Supplementary File S2: Adapted AGREE II instrument used in this study; Supplementary File S3: Results of completed AGREE II appraisals; Supplementary File S4: Details of excluded studies with reasons for exclusion; Supplementary File S5: PRISMA 2020 checklist; Supplementary File S6: Characteristics of studies included in the present systematic review; Supplementary File S7 (Figures S1–S10): Risk of bias (RoB 2) assessments for each included study (LoE II and III) and AMSTAR 2 confidence ratings for included existing published systematic reviews and meta-analyses.
