# Supplementary material for: Photobiomodulation Therapy in the Management of Orofacial Neuropathic Pain—WALT Position Paper 2026
Source: J Clin Med. 2026 Feb 6;15(3):1304. doi: 10.3390/jcm15031304 (PMC12898000; doi:10.3390/jcm15031304)
Supplement: Supplementary file 1 [file jcm-15-01304-s001.zip › Supplementary File S1-AMSTAR 2.pdf]

AMSTAR 2 Critical Appraisal Checklist for Systematic Reviews that utilised for this Position Paper.
Critical Domains as follows:

These are: 2, 4, 7, 9, 11, 12, 13

Multiple flaws in these can lower confidence to **low** or **critically low**.

	**Criterion**	**Yes / No / Partial**	**Comments**
1	Did the research questions and inclusion criteria include the components of PICO (Population, Intervention, Comparator, Outcomes)?		
2	Was the review protocol established before conducting the review, and were deviations justified?		
3	Did the authors explain their selection of study designs for inclusion?		
4	Was a comprehensive literature search strategy used?		
5	Was study selection performed in duplicate?		
6	Was data extraction performed in duplicate?		
7	Did the authors provide a list of excluded studies and justify the exclusions?		
8	Were the included studies described in adequate detail?		
9	Was the risk of bias in individual studies adequately assessed?		
10	Did the authors report on funding sources for included studies?		
11	If meta-analysis was performed, were appropriate methods used for combining results?		
12	If meta-analysis was performed, did the authors assess the impact of RoB on the meta-analysis results?		
13	Did the authors account for RoB in interpreting/discussing results?		
14	Was heterogeneity explained and discussed adequately?		
15	If quantitative synthesis was done, was publication bias investigated and discussed?		
16	Did the authors report conflicts of interest and funding for the review itself?		
