# Supplementary material for: Photobiomodulation Therapy in the Management of Orofacial Neuropathic Pain—WALT Position Paper 2026
Source: J Clin Med. 2026 Feb 6;15(3):1304. doi: 10.3390/jcm15031304 (PMC12898000; doi:10.3390/jcm15031304)
Supplement: Supplementary file 1 [file jcm-15-01304-s001.zip › Supplementary File S2-Adapted AGREE II checklist .pdf]

Adapted AGREE II checklist for the context of PBM in the management of neuropathic pain in orofacial conditions and was used to support confirmation that WALT recommendations are justified.

Domain 1: Scope and Purpose

AGREE Focus	How to Adapt	Page
**Item 1- Objectives**	Clearly state that your objective is to evaluate and propose PBM protocols for managing neuropathic orofacial pain	
**Item 2- Health Questions**	Specify clinical questions like: "What PBM parameters are effective for neuropathic orofacial pain?" or "What is the recommended dosimetry for different conditions?	
**Item 3- Population**	Define the patient population clearly (e.g., adults with clinically diagnosed neuropathic orofacial pain, ICD-11 criteria, etc.). Include any subgroups if relevant.	

Domain 2: Stakeholders Involvement

AGREE Focus	How to adapt	Page
**Item 4 – Guideline Development Group**	List contributors including PBM experts, pain specialists, dentists, and methodologists. Mention multidisciplinary input.	
**Item 5 – Target Population Preferences**	If available, include patient-reported outcomes, preferences, or qualitative data (e.g., tolerance to treatment, ease of application).	
**Item 6 – Target Users**	Define that the protocol is aimed at clinicians, dentists, physiotherapists, or researchers applying PBM in neuropathic pain care.	

Domain 3: Rigour of Development

AGREE Focus	How to Adapt	Page
**Item 7 – Evidence Search**	Describe your literature search strategy, databases, date range, and inclusion/exclusion criteria (e.g., focusing on RCTs and low risk of bias studies).	
**Item 8 – Criteria for Selecting Evidence**	Clearly explain how you assessed study quality (e.g., using GRADE and risk of bias tools).	
**Item 9 – Evidence Strength**	Summarise strengths and limitations of evidence for PBM effectiveness and its benefits per condition	
**Item 10-Safety**	Summarises strengths and limitations of the evidence for PBM effectiveness and its benefits for each condition.	
**Item 11 – Formulating Recommendations**	State how dosimetry recommendations were derived (e.g., median parameters from studies with positive outcomes).	
**Item 12 – External Review**	Indicate if the protocol was reviewed by external experts, professional societies, or WALT members.	
**Item 13 – Updating**	Mention a plan to revise the protocol as new PBM evidence becomes available.	

Domain 4: Clarity on PBM dosimetry and treatment protocols Recommendations

AGREE Focus	How to adapt	Page
**Item 14 – Recommendations Specific and Unambiguous**	Clearly list PBM parameters (wavelength, power density, duration, frequency, treatment site) per condition. Use tables or algorithms.	
**Item 15 – Management Options**	Describe different PBM protocols if variability exists (e.g., low vs. moderate energy density).	
**Item 16 – Key Recommendations Easily Identified**	Highlight main treatment protocols (e.g., shaded boxes, summary tables, or flowcharts).	

Domain 5: Applicability

AGREE Focus	How to adapt	Page
**Item 17 – Implementation Tools**	Provide tables, dosing calculators, or visual guides to help clinicians apply the protocols.	
**Item 18 – Barriers/ Facilitators**	Discuss challenges like device availability, training, or standardisation.	
**Item 19 – Resource Implications**	Briefly outline cost considerations or device options for PBM. Summarises any reported cost-effectiveness data of PBM interventions for each condition.	
**Item 20 – Monitoring/Audit Criteria**	Suggest clinical outcome measures (e.g., VAS scores, pain diaries) for tracking PBM effectiveness.	

Domain 6: Editorial Independence

AGREE Focus	How to adapt	Page
**Item 21 – Funding Body Influence**	Disclose if the protocol was funded and clarify that funders had no influence over recommendations.	
**Item 21 – Competing Interests**	Include conflict of interest disclosures for all authors.	
