# Supplementary material for: Photobiomodulation Therapy in the Management of Orofacial Neuropathic Pain—WALT Position Paper 2026
Source: J Clin Med. 2026 Feb 6;15(3):1304. doi: 10.3390/jcm15031304 (PMC12898000; doi:10.3390/jcm15031304)
Supplement: Supplementary file 1 [file jcm-15-01304-s001.zip › Supplementary File S3-Completed AGREE II checklist.pdf]

Adapted AGREE Reporting Checklist for this Position Paper: Best Practice Guidelines and Recommendations

Domain	AGREE Focus	How to Adapt	Page for each condition		
			BMS	TN	PHN
**Domain 1:** Scope and Purpose	Item 1- Objectives	Clearly state that your objective is to evaluate and propose PBM protocols for managing neuropathic orofacial pain	9, 10	10,11	10,12
	Item 2- Health Questions	Specify clinical questions like: "What PBM parameters are effective for neuropathic orofacial pain?" or "What is the recommended dosimetry for different conditions?	10,11	11	12
	Item 3- Population	Define the patient population clearly (e.g., adults with clinically diagnosed neuropathic orofacial pain, ICD-11 criteria, etc.). Include any subgroups if relevant.	Supplementary File #6	Supplementary File #6	Supplementary File #6
**Domain 2:** Stakeholders Involvement	Item 4 – Guideline Development Group	List contributors including PBM experts, pain specialists, dentists, and methodologists. Mention multidisciplinary input.	multidisciplinary input.	multidisciplinary input.	multidisciplinary input.
	Item 5 – Target Population Preferences	If available, include patient-reported outcomes, preferences, or qualitative data (e.g., tolerance to treatment, ease of application).	11	11	12
	Item 6 – Target Users	Define that the protocol is aimed at clinicians, dentists, physiotherapists, or researchers applying PBM in neuropathic pain care.	11	11	12
**Domain 3:** Rigour of Development	Item 7 – Evidence Search	Describe your literature search strategy, databases, date range, and inclusion/exclusion criteria (e.g., focusing on RCTs and low risk of bias studies).	6,9,10, Supplementary File #4 ,6,7	6,9,10, Supplementary File #4 ,6,7	6,9,10, Supplementary File #4 ,6,7
	Item 8 – Criteria for Selecting Evidence	Clearly explain how you assessed study quality (e.g., using GRADE and risk of bias tools).	5 9,10; GRADE, Level of Evidence; RoB, ROBINS I; AMSTAR 2	5,9,10; GRADE, Level of Evidence; RoB, ROBINS I; AMSTAR 2	5,9,10; GRADE, Level of Evidence; RoB, ROBINS I; AMSTAR 2
	Item 9 – Evidence Strength	Summarise strengths and limitations of evidence for PBM effectiveness and its benefits per condition	Supplementary File #6	Supplementary File #6	Supplementary File #6
	Item 10-Safety	Summarises strengths and limitations of the evidence for PBM effectiveness and its benefits for each condition.	Supplementary File #6	Supplementary File #6	Supplementary File #6
	Item 11 – Formulating Recommendations	State how dosimetry recommendations were derived (e.g., median parameters from studies with positive outcomes).	10,11	11,12	12,13
	Item 12 – External Review	Indicate if the protocol was reviewed by external experts, professional societies, or WALT members.	1,7,8	1,7,8	1,7,8
	Item 13 – Updating	Mention a plan to revise the protocol as new PBM evidence becomes available.	13	13	13
**Domain 4:** Clarity on PBM dosimetry and treatment protocols Recommendations	Item 14 – Recommendations Specific and Unambiguous	Clearly list PBM parameters (wavelength, power density, duration, frequency, treatment site) per condition. Use tables or algorithms.	11	11	12
	Item 15 – Management Options	Describe different PBM protocols if variability exists (e.g., low vs. moderate energy density).			
	Item 16 – Key Recommendations Easily Identified	Highlight main treatment protocols (e.g., shaded boxes, summary tables, or flowcharts).	11	11	12
**Domain 5:** Applicability	Item 17 – Implementation Tools	Provide tables, dosing calculators, or visual guides to help clinicians apply the protocols.	11	11	12
	Item 18 – Barriers/ Facilitators	Discuss challenges like device availability, training, or standardisation.	13,14	13,14	13,14
	Item 19 – Resource Implications	Briefly outline cost considerations or device options for PBM.<br>Summarises any reported cost-effectiveness data of PBM interventions for each condition.	NA	NA	NA
	Item 20 – Monitoring/Audit Criteria	Suggest clinical outcome measures (e.g., VAS scores, pain diaries) for tracking PBM effectiveness.	11	11	11

**Domain 6:** Editorial Independence	Item 21 – Funding Body Influence	Disclose if the protocol was funded and clarify that funders had no influence over recommendations.	14	14	14
	Item 21 – Competing Interests	Include conflict of interest disclosures for all authors.	14	14	14
