# Supplementary material for: Photobiomodulation Therapy in the Management of Orofacial Neuropathic Pain—WALT Position Paper 2026
Source: J Clin Med. 2026 Feb 6;15(3):1304. doi: 10.3390/jcm15031304 (PMC12898000; doi:10.3390/jcm15031304)
Supplement: Supplementary file 1 [file jcm-15-01304-s001.zip › Supplementary File S4-excluded studies.pdf]

Articles excluded due to the review criteria

1. Seada, Y.I.; Nofel, R.; Sayed, H.M. Comparison between transcranial electromagnetic stimulation and low-level laser on modulation of trigeminal neuralgia. *J Phys Ther Sci.* **2013**, *25*, 911–914. Https:// doi.org/10.1589/jpts.25.911
2. Amanat, D.; Ebrahimi, H.; Lavaee, F.; Alipour. A.The adjunct therapeutic effect of lasers with medication in the management of orofacial pain: double blind randomized controlled trial. *Photomed Laser Surg*. **2013**, *31*, 474–479. https://doi.org/10.1089/pho.2013.3555
3. Sessirisombat, S. Carbon-dioxide laser in the treatment of trigeminal neuralgia: a preliminary study. *J Interdiscipl Med Dent Sci*. **2017**, 5, 208.
4. Walker, J. Relief from chronic pain by low power laser irradiation. *Neurosci Lett,* **1983**, 43, 339–344. https://doi.org/10.1016/0304- 3940(83)90211-2 (combined cohort)
5. Antonic´, R.; Brumini, M.; Vidovic, I.; Urek, M.M.; Glazˇar, I.; Pezelj- Ribaric, S. The effects of low level laser therapy on the management of chronic idiopathic orofacial pain: Trigeminal neuralgia, temporomandibular disorders and burning mouth syndrome. *Medicina Fluminensis* **2017**, *53*, 61–67.
6. Kim HK, Jung JH, Kim CH et al., The Effect of Lower Level Laser Therapy on Trigeminal Neuralgia (Chinese script)
7. Moore, C.K.; hira, N.; Kumar, P.S.; Jayakumar, C.S. et al., A double blind crossover trial of low level laser therapy in the treatment of postherpetic neuralgia. *Laser Therapy* Pilot Edition 1:0, **1998**
8. Wang, Q.; Ye, Y.; Yang, L. et al. Painful diabetic neuropathy: The role of ion channels. *Biomed Pharmacother*. **2024**, *173*, 116417. doi:10.1016/j.biopha.2024.116417
9. Falaki, F.; Nejat, A.H.; Dalirsani, Z. The Effect of Low-level Laser Therapy on Trigeminal Neuralgia: A Review of Literature. *J Dent Res Dent Clin Dent Prospects*. **2014**, *8*, 1-5. http:// doi:10.5681/joddd.2014.001
10. Aguiar, G.A.; Pinheiro, A.L.B.; Cangussu, M.C. Use of Laser Phototherapy in The Treatment of Trigeminal Neuralgia. *Int j health & medical research*. **2024**, 3, 121-126. doi: 10.58806/ijhmr.2024.v3i3n05
11. Mukhtar, R.; Fazal, M.U.; Saleem, M.; Saleem, S. Role of low-level laser therapy in post-herpetic neuralgia: a pilot study. *Lasers Med Sci*. **2020**, *35*, 1759-1764. doi:10.1007/s10103-020-02969-5
12. Eduardo, P.C.; Aranha, A.C.; Simões, A.; Bello-Silva, M.S.; Ramalho, K.M.; Esteves-Oliveira, M.; de Freitas, P.M.; Marotti, J.; Tunér, J. Laser treatment of recurrent herpes labialis: a literature review. *Lasers Med Sci.* **2014**, *29*, 1517-29. doi: 10.1007/s10103-013-1311-8.
13. Zanella PA, Onuchic LF, Watanabe EH, Azevedo LH, Aranha ACC, Ramalho KM, Eduardo CP. Photobiomodulation for Preventive Therapy of Recurrent Herpes Labialis: A 2-Year *In Vivo* Randomized Controlled Study. *Photobiomodul Photomed Laser Surg*. **2022**, *40*, 682-690. doi: 10.1089/photob.2022.0054.
14. Merrick R, Kahn F, Saraga F. Treatment of Postherpetic Neuralgia With Low Level Laser Therapy. Pract Pain Manag. 2013;13G6I.
15. Mahdi, A.S. Low level of laser therapy (lllt) of laser diode 820nm in the treatment trigeminal neuralgia. *Laser in Medicine*, **2014**, *1*, 1-8.
16. Spadari, F.; Pulicari, F.; Ghizzoni, M.; Porrini, M.; Bosotti, M.; Pellegrini, M. Photobiomodulation as a Therapeutic Strategy in Burning Mouth Syndrome: A Scoping Review. *Applied Sciences*. **2023**, *13*, 8880. https://doi.org/10.3390/app13158880
17. Ravera, S.; Colombo, E.; Pasquale, C.; Benedicenti, S.; Solimei, L.; Signore, A.; Amaroli A. Mitochondrial Bioenergetic, Photobiomodulation and Trigeminal Branches Nerve Damage, What's the Connection? A Review. *Int J Mol Sci.* **2021**, *22*, 4347. doi: 10.3390/ijms22094347.
18. Sharifi, R.; Fekrazad, R.; Taheri, M.M., Kasaeian, A.; Babaei, A. Effect of photobiomodulation on recovery from neurosensory disturbances after sagittal split ramus osteotomy: A triple-blind

randomised controlled trial. *Br. J. Oral Maxillofac. Surg.* **2020**, *58*, 535–541. doi: 10.1016/j.bjoms.2020.02.005.
19. De Oliveira, R.F.; Da Silva, A.C.; Simões, A.; Youssef, M.N.; De Freitas, P.M. Laser Therapy in the Treatment of Paresthesia: A Retrospective Study of 125 Clinical Cases. *Photomed. Laser Surg*. 2015, 33, 415–423. doi: 10.1089/pho.2015.3888.
20. Esteves Pinto Faria P., Temprano A., Piva F., Sant'ana E., Pimenta D. Low-level laser therapy for neurosensory recovery after sagittal ramus osteotomy. Minerva Stomatol. 2020;69:141–147. doi: 10.23736/S0026-4970.20.04289-2. [DOI] [PubMed] [Google Scholar]
21. Guarini D., Gracia B., Ramírez-Lobos V., Noguera-Pantoja A., Solé-Ventura P. Laser Biophotomodulation in Patients with Neurosensory Disturbance of the Inferior Alveolar Nerve after Sagittal Split Ramus Osteotomy: A 2-Year Follow-Up Study. Photomed. Laser Surg. 2018;36:3–9. doi: 10.1089/pho.2017.4312.

In vitro/Animal studies

1. De Andrade ALM, Bossini PS, do Canto De Souza ALM, Sanchez AD, Parizotto NA. Effect of photobiomodulation therapy (808 nm) in the control of neuropathic pain in mice. *Lasers Med Sci*. 2017;32(4):865-872. Doi:10.1007/s10103-017-2186-x
2. Chacur M, Rocha IRC, Harland ME, et al. Prevention and reversal of neuropathic pain by near-infrared photobiomodulation therapy in male and female rats. *Physiol Behav*. 2024;286:114680. doi:10.1016/j.physbeh.2024.114680
3. de Oliveira ME, Da Silva JT, Brioschi ML, Chacur M. Effects of photobiomodulation therapy on neuropathic pain in rats: evaluation of nociceptive mediators and infrared thermography. *Lasers Med Sci*. 2021;36(7):1461-1467. doi:10.1007/s10103-020-03187-9
4. Li F, Fang L, Huang S, et al. Hyperbaric oxygenation therapy alleviates chronic constrictive injury-induced neuropathic pain and reduces tumor necrosis factor-alpha production. *Anesth Analg*. 2011;113(3):626-633. doi:10.1213/ANE.0b013e31821f9544
5. Holanda VM, Chavantes MC, Wu X, Anders JJ. The mechanistic basis for photobiomodulation therapy of neuropathic pain by near infrared laser light. *Lasers Surg Med*. 2017;49(5):516-524. doi:10.1002/lsm.22628
6. Micheli L, Di Cesare Mannelli L, Lucarini E, et al. Photobiomodulation therapy by NIR laser in persistent pain: an analytical study in the rat. *Lasers Med Sci*. 2017;32(8):1835-1846. doi:10.1007/s10103-017-2284-9
7. Moradi A, Ghaffari Novin M, Bayat M. A Comprehensive Systematic Review of the Effects of Photobiomodulation Therapy in Different Light Wavelength Ranges (Blue, Green, Red, and Near-Infrared) on Sperm Cell Characteristics in Vitro and in Vivo. *Reprod Sci*. 2024;31(11):3275-3302. doi:10.1007/s43032-024-01657-x

Case report/case series

1. Gomes R, Vianna L, Ramos J, et al. Effects of photobiostimulation in the treatment of post-herpetic neuralgia: a case report. Rev. Bras. Geriatr. Gerontol., Rio de Janeiro, 2018; 21(1): 102-107. http://dx.doi.org/10.1590/1981-22562018021.170116
2. l-Kufi, H., Alhumadi, A., Ali, M. *et al.* Therapeutic and analgesic efficacy of Photobiomodulation Therapy (PBMT) in the management of trigeminal neuralgia: a case report. *Laser Dent Sci* 8, 55 (2024). https://doi.org/10.1007/s41547-024-00270-0
3. Vernon L, Hasbun R. Low-level Laser Therapy for Trigeminal Neuralgia. Pract Pain Manag. 2008;8(6).
4. Gopal S, Kumar M, Harini BK. Low level laser therapy in the management of trigeminal neuralgia: A rare case report. International Journal of Science and Research Archive, 2024, 12(01), 2026–2030. https://doi.org/10.30574/ijsra.2024.12.1.0938

5. Bouguezzi A, Garma M. The effect of low-level laser therapy on persistent Idiopathic facial pain. *J Pain Manage Ther 2021, 5(4), 1-3.*
6. Derikvand N, Hatami M, Ghasemi S, Fallahnia N. Photobiomodulation as a Coadjutant in Management of Trigeminal Neuralgia: A Case Series Study and Review of the Literature. Journal of "Regeneration, Reconstruction & Restoration"(Triple R). 2024; Volume 9:e2. Doi: 10.22037/rrr.v9.38696
7. Tanganeli J, Haddad D, Bussadori S. Photobiomodulation as an adjuvant in the pharmacological treatment of trigeminal neuralgia. Case report. BrJP. São Paulo, 2020;3(3):285-7. DOI 10.5935/2595-0118.20200042
8. Demchak T, Carraway C. Using Photobiomodulation to Treat Trigeminal Neuralgia. Pract Pain Manag. 2020;20(6).
9. Sadrabad M, Pedram A, Saberian E, et al. Clinical efficacy of LLLT in treatment of trigeminal neuralgia – Case report. Journal of Oral and Maxillofacial Surgery, Medicine, and Pathology. 2023, 35(6), 568-571. https://doi.org/10.1016/j.ajoms.2023.03.012
10. Martines, D.M.; Huh, B.K.; Javed, S. Case Reprot: Use of High-Intesnity Laser Therapy for Treatmetn of Trigemianl Neuralgia. Pain Management, 2023, 13, 709-716.
11. Fu, Jing MD[a]; Nie, Zhiqiang MM[b]; Zhang, Yanfeng, Tang, Min, Guo, Jianguo . A retrospective comparative case series of efficacy and safety of radiofrequency thermocoagulation versus drug therapy in patients with trigeminal neuralgia: A clinical case report. Medicine 103(38):p e39353, September 20, 2024
12. Cervo E, Boneti M, Scolari n et al. Effect of photobiomodulation on pain perception in burning mouth syndrome: a clinical case report. 2025, 139 (5), e75
13. De Freitas Rodrigues A., De Oliveira Martins D., Chacur M., Luz J.G.C. The effectiveness of photobiomodulation in the management of temporomandibular pain sensitivity in rats: Behavioral and neurochemical effects. Lasers Med. Sci. 2020;35:447–453. doi: 10.1007/s10103-019-02842-0.
14. Desiderá A.C., Nascimento G.C., Gerlach R.F., Leite-Panissi C.R. Laser therapy reduces gelatinolytic activity in the rat trigeminal ganglion during temporomandibular joint inflammation. Oral Dis. 2015;21:652–658. doi: 10.1111/odi.12330.
15. Diker N., Aytac D., Helvacioglu F., Oguz Y. Comparative effects of photobiomodulation therapy at wavelengths of 660 and 808 nm on regeneration of inferior alveolar nerve in rats following crush injury. Lasers Med. Sci. 2020;35:413–420. doi: 10.1007/s10103-019-02838-w.
16. Hakimiha N., Dehghan M.M., Manaheji H., Zaringhalam J., Farzad-Mohajeri S., Fekrazad R., Moslemi N. Recovery of inferior alveolar nerve by photobiomodulation therapy using two laser wavelengths: A behavioral and immunological study in rat. J. Photochem. Photobiol. B. 2020;204:111785. doi: 10.1016/j.jphotobiol.2020.111785. [DOI] [PubMed] [Google Scholar]
17. Martins D.O., Dos Santos F.M., Ciena A.P., Watanabe I.S., De Britto L.R.G., Lemos J.B.D., Chacur M. Neuropeptide expression and morphometric differences in crushed alveolar inferior nerve of rats: Effects of photobiomodulation. Lasers Med. Sci. 2017;32:833–840. doi: 10.1007/s10103-017-2181-2. [DOI] [PubMed] [Google Scholar]
18. Miloro M., Halkias L.E., Mallery S., Travers S., Rashid R.G. Low-level laser effect on neural regeneration in Gore-Tex tubes. Oral Surg. Oral Med. Oral Pathol. Oral Radiol. Endodontol. 2002;93:27–34. doi: 10.1067/moe.2002.119518. [DOI] [PubMed] [Google Scholar]
19. Noma D., Fujita S., Zama M., Mayahara K., Motoyoshi M., Kobayashi M. Application of oxytocin with low-level laser irradiation suppresses the facilitation of cortical excitability by partial ligation of the infraorbital nerve in rats: An optical imaging study. Brain Res. 2020;1728:146588. doi: 10.1016/j.brainres.2019.146588. [DOI] [PubMed] [Google Scholar]
20. Sasaki R.T., Grossi N.G., Zeni R.T., Saez D.M., Gonçalves I.D., Da Silva M.C.P. Effect of Laser Photobiomodulation with Gradual or Constant Doses in the Regeneration of Rats' Mental Nerve After Lesion by Compression. Photomed. Laser Surg. 2017;35:408–414. doi: 10.1089/pho.2016.4210. [DOI] [PubMed] [Google Scholar]

21. Yucesoy T., Kutuk N., Canpolat D.G., Alkan A. Comparison of Ozone and Photo-Biomodulation Therapies on Mental Nerve Injury in Rats. J. Oral Maxillofac. Surg. 2017;75:2323–2332. doi: 10.1016/j.joms.2017.04.016. [
22. Buchaim D.V., Rodrigues Ade C., Buchaim R.L., Barraviera B., Junior R.S., Junior G.M., Bueno C.R., Roque D.D., Dias D.V., Dare L.R., et al. The new heterologous fibrin sealant in combination with low-level laser therapy (LLLT) in the repair of the buccal branch of the facial nerve. Lasers Med. Sci. 2016;31:965–972. doi: 10.1007/s10103-016-1939-2.
23. Kato, I. T.; Pellegrini, V. D.; Prates, R. A.; Ribeiro, M. S.; Wetter, N. U.; Sugaya, N. N. Low-level laser therapy in burning mouth syndrome patients: a pilot study. *Photomed Laser Surg*, **2010**, *28*, 835–839. https://doi.org/10.1089/pho.2009.2630
24. dos Santos, L.deF.; Carvalho, A.deA.; Leão, J. C.; Cruz Perez, D. E.; Castro, J. F. Effect of low-level laser therapy in the treatment of burning mouth syndrome: a case series. *Photomed Laser Surg*, **2011**, *29*, 793–796. https://doi.org/10.1089/pho.2011.3016
