# Supplementary material for: Photobiomodulation Therapy in the Management of Orofacial Neuropathic Pain—WALT Position Paper 2026
Source: J Clin Med. 2026 Feb 6;15(3):1304. doi: 10.3390/jcm15031304 (PMC12898000; doi:10.3390/jcm15031304)
Supplement: Supplementary file 1 [file jcm-15-01304-s001.zip › Supplementary File S6-included studies in SR.pdf]

Twenty-six studies on Primary Burning Mouth Syndrome (BMS)-induced NP were deemed eligible for inclusion in the systematic review for the Position Paper Recommendations.

First author, Year and Country	Study characteristics	Study Groups	Follow up	PBMT parameters								Overall Risk of bias {RoB: Low, Some concerns, High] [ROBINS: Low, Moderate, Serious]	Level of Evidence [Melnyk B et al., 2011]	Outcome
				λ (nm)	Power (mW)	Irradiance (W/cm^2)	Energy (J)/point	Fluence J/cm^2	Beam area (cm^2)	Irradiation time (s)	Frequency per week/ Rx duration			
Hanna et al., 2022, UK (London) [11]	Human, Non-randomised study, controlled, 28 participants (28 completed)	Groups:<br>- PBM<br>-Pharmacotherapy	Up to 9 months	810	200	1.97	6<br>Total 54 J	59.1	0.088 cm^2	30 /point	Twice a week/ 5 consecutive weeks	Low	III	Results showed that despite the severe and persistent nature of the symptoms of 57.50 ± 47.93 months at baseline in the PBM group, a notably rapid reduction in PISmax on VAS from 7.6 at baseline (T0) to 3.9 at one-month post-treatment (T3) could be achieved. On the other hand, mean PISmax was only reduced from 8.2 at baseline to 6.8 at T3 in the MED [pharmacotherapy] group<br>Comment: The parameters were clearly reported
de Pedro et al., 2020, Spain (Madrid) [40]	Human Randomised trial, sham-controlled, 20 participants	LLLT vs sham	> 4 month	810	600	1.2	6	12	0.5 cm^2	10	Twice a week / 5 weeks (total 10 sessions	Low	II	Photobiomodulation seems to be effective in reducing pain in patients with BMS, as well as, having a positive impact on the psychological state of these patients.
Bardellini et al., 2019, Italy [41]	Human Randomised trial, double-blind, 90 participants (85 completed)	PBM vs placebo	> 1 month	660, 800,970	Medium 3200 (6400 mW pulsed 50%)	3.2	-	-	1 cm^2	231	10-week treatment	Low	II	Patients treated with PBMT showed a significant decrease in symptoms (p=0.0008) and improved quality of life related to oral health (p=0.0002).<br>Comment: The differences between the groups became significant after the 6th session. It's positive that this study measured the outcomes after each session
Sugaya et al., 2016, Brazil (São Paulo) [42]	Human Randomised trial, sham-controlled, 30 participants (23 completed)	Groups:<br>- laser<br>- placebo	90 days	790	120	4	6/ point (number of points varied)	6	0.03 cm^2	50 /point	4 / 2 weeks	Low	II	According to the protocol used in this study, low intensity laser therapy is as beneficial to patients with BMS as placebo treatment, indicating a great emotional component of involvement in BMS symptomatology. Nevertheless, there were positive results in some statistical analyses, thus encouraging further research in BMS laser therapy with other irradiation parameters.
Arduino et al., 2016, Italy (Turin) [43]	Human Comparison study, randomised, 33 participants (33 completed), PBM vs clonazepam	Groups:<br>- LLLT<br>- clonazepam	12 weeks	980	300	1	3	10	0.28 cm^2	10 /point	Twice a week / 5 weeks (total 10 sessions)	Low	II	Comparing the two groups, LLLT appeared to be superior in improving pain perception, but statistically only at 8 weeks after the end of the protocol proposed (P = 0.026).<br>Comment: No negative control group, but a positive control group (clonazepam).
Lu et al., 2025, China (Shanghai) [44]	Human Randomised trial, sham-controlled, Multicentre, 128 participants (123 completed)	Groups:<br>- PBM<br>- cryotherapy<br>- combination<br>- drug therapy	7-week treatment / 12-week study	633	25	0.050	15 (10p)	3	0.5 cm^2	600	7 / 7 weeks	Some concerns	II	After 7 weeks of treatment, the PBM + OCT group achieved a high overall response rate for pain reduction (81.25%). This difference in pain reduction trends between the groups resulted in a nearly fivefold greater mean change in the VAS score at the 12-week assessment for the PBM + OCT group than for the DT group (p < 0.0083). Furthermore, anxiety symptoms were also significantly alleviated by PBM combined with OCT, resulting in a nearly tenfold greater mean change in the GAD-7 score at the 7-week assessment in the PBM + OCT group than in the DT group (p < 0.0083). No severe adverse events were reported during the study period
Ge et al., 2025, China (Shanghai) [45]	Human Comparison study, randomised, 63 participants (53 completed)	Wavelength comparison<br>Groups:<br>- 660 nm<br>- 810 nm<br>- 975 nm	4 weeks	660<br>810<br>975	50<br>500<br>30	0.050<br>0.500<br>0.030	12.75<br>18<br>60/70	1.5<br>3<br>10	1 cm^2	30,<br>6,<br>33 /point (43 points)	4 / 4 weeks	Some concerns	II	Statistically significant pain relief was observed in all groups (median reduction of 40%). The improvement of numbness in the 810 nm and 975 nm groups (median reduction of 40%) was also significant (p < 0.05). However, no significant differences in efficacy were noted among the three groups (p > 0.05). These results suggested that LLLT with three different wavelengths effectively reduced pain and that the 810 nm and 975 nm wavelengths also significantly alleviated numbness in BMS patients
Martinez et al., 2024, Spain (Murcia) [46]	Human Randomised trial, sham-controlled, 89 participants PBM vs clonazepam	Groups:<br>- PBM + clonazepam<br>- sham PBM<br>- PBM<br>-clonazepam	1 month	-	-	-	-	6	19 mm^2	30 / site	4 / 4 weeks	Some concerns	II	A significant decrease in the VAS scores was observed after treatment in group 1 (laser + clonazepam) (p = 0.029) and group 3 (laser) (p = 0.005). The low-level diode laser is a good treatment option in BMS, resulting in a decrease in patient symptoms and in salivary biomarkers. However, standardization of the intervention protocols and laser intensity parameters is needed in order to draw more firm conclusions. Comment: Parameters inadequately reported.
Barbosa et al., 2018, Brazil (Natal) [47]	Human Comparison study, randomised, 44 participants (33 completed)	LLLT vs alpha-lipoic acid<br>Groups:<br>- burning mouth / laser<br>- burning mouth / ALA	4 weeks	660	30	-	-	3	⌀ = 3 mm	10 /site	4 / 4 weeks	Some concerns	II	The results of this study suggest that LLLT and ALA are efficient therapies in reducing burning mouth symptoms, with LLLT being more efficient than ALA.<br>Comment: Uneven randomizing: "BMS/laser (n = 10), BMS/ALA (n = 5), SOB/laser (n = 15), and SOB/ALA (n = 14).

		- sec. oral burn, / laser<br>- sec. oral burn. / ALA												
Arbabi-Kalati et al.,2015, Iran (Zahedan)<br>[48]	Human<br>Randomised trial, sham-controlled, 20 participants	Groups:<br>- laser<br>- placebo	4 weeks	630	30	-	-	1	-	10	8<br>/ 4 weeks	Some concerns	II	Burning sensation severity and quality of life in the two groups after intervention were different significant statistically, ($p= 0.004$, $p= 0.01$ respectively) .Patients in laser group had better results.<br>Comment: Parameters inadequately reported.
Medeiros et al., 2023, Brazil, (Natal)<br>[49]	Human<br>Comparison study, 25 participants	Groups:<br>- PBM<br>- TENS	8-week treatment -> 30-day follow-up	808	100	3.33	6<br>/point<br><br>(44 points)	200	0.03 cm^2	60<br>/point<br><br>(44 points)	8<br>/ 8 weeks	High	II	TENS and PBM were effective in reducing the symptoms of burning mouth during and after treatment. The PBM group showed a better response during follow-up<br>Comment: On the other hand, TENS was slightly better at the end of treatment.
Lončar-Brzak et al., 2022, Croatia (Zagreb)<br>[50]	Human<br>Randomised trial, 80 participants (62 completed)	PBM vs passive vs vitamin B vs probiotics	2-week treatment -> 1-month follow-up	685	30	0.003	-	2	3 cm^2	381	10<br>/ 2 weeks	High	II	Comment: Hard to interpret the study since the baseline symptom score was different in the passive group (information only) compared to active groups.
Scardina et al., 2020, Italy (Palermo)<br>[51]	Human<br>Randomised trial, sham-controlled, 40 participants		4-week treatment -> 60-day follow-up	800	60	0.180	1200	50	-	1200<br>(4 sites)	8<br>/ 4 weeks	High	II	The group of patients who underwent laser therapy, there was a lasting improvement in symptoms. The capillary oral bed of patients in the placebo group did not show any statistically significant difference ($p > 0.05$). In the laser group we observed the following: in the buccal mucosa the diameter of the capillary had a reduction of 3 µm; in the upper lip mucosa, there was a reduction of 3 µm; in the lower lip mucosa, there was a reduction of 3 µm; and in the dorsal lingual surface, there was a reduction of 2 µm. An increase in capillary length was also obtained in all irradiated regions in the laser group patients ($p < 0.05$). PBM induces microcirculatory changes that are still present over a long period of time, such as an improvement in the clinical picture. The improvement in the symptoms has been correlated to the reduction of the capillary diameter. The placebo effect only led to a temporary improvement in symptoms that were unrelated to changes in the microcirculatory pattern.<br>Comment: Abstract says 800 nm, full text says 805 nm wavelength.
Škrinjar et al., 2020, Croatia (Zagreb)<br>[52]	Human<br>Randomised trial, double-blind, 23 participants (23 completed)		2 weeks	685	30	0.003	-	2	3 cm^2	381	10<br>/ 2 weeks	High	II	VAS scores and salivary cortisol levels were significantly lower in both groups after LLLT.<br>Comment: PBM was not better than placebo, yet still the abstract makes it seem like PBM was effective.
Spanemberg et al., 2019, Spain<br>[53]	Human<br>Randomised trial, 21 participants (21 completed)		4-week treatment -> 2-month follow-up	808	200	1.97	3<br>/point	-	0.088 cm^2	15<br>/point	8<br>/ 4 weeks	High	II	The initial VAS score mean was 8.9 for the LG and 8.3 for the CG ($p > 0.05$). After the eighth session the VAS score was 5.5 and 5.8 respectively, and at two months it was 4.7 and 5.1 respectively. The improvement was marginally significant in the multivariant analysis of: dry mouth, dysgeusia, pain and the treatment ($p=0.0538$).
Sikora et al., 2018, Croatia (Osijek)<br>[54]	Human<br>Randomised trial, sham-controlled 44 participants		2 weeks	830	-	-	-	12	1 cm^2	300<br><br>(session duration)	10<br>/ 2 weeks	High	II	There were no significant differences between the groups before and after LLLT (switched on and off) in the quality of life (OHIP CRO 14 scores) ($p>0.05$). There was significant decrease in pain symptoms (VAS) in both LLLT switched on and LLLT switched off groups ($p < 0.05$). Both LLLT switched on and switched off decreased pain symptoms (VAS) in patients with BMS; however, neither LLLT switched off or switched on im-proved the OHIP-CRO 14 scores.
Spanemberg et al., 2015, Brazil<br>[55]	Human<br>Randomised trial, 78 participants (78 completed)	Wavelength comparison<br>Groups:<br>- 830nm 1/wk, 10 total<br>- 830nm 3/wk, 9 total<br>- 685nm 3/wk, 9 total<br>- control group	3-week or 10-week treatment -> 8-week follow-up	685<br><br>830	35<br><br>100	1.25<br><br>3.57	2/point<br><br>5/point	72<br><br>172	0.028 cm^2	58/p<br><br>50/p	10<br>/ 10 weeks<br><br>or<br><br>9<br>/ 3 weeks	High	II	There was significant reduction of the symptoms in all groups at the end of the treatment, which was maintained in the follow-up. The scores of the IR1W and IR3W laser groups differed significantly from those of the CG.
Pezelj-Ribarić et al., 2013, Croatia<br>[56]	Human Non-randomsed study, sham-controlled, 40 participants	Groups:<br>- LLLT<br>- placebo laser	4 weeks	685	30	-	-	3	2 mm	600	20<br>/ 4 weeks	High	II	LLLT did not have beneficial effects in this study.<br>Comment: The study says participants were "divided" into groups but it is not mentioned whether they were randomly divided or whether there was some other allocation method. Thus it's interpreted that this study is not randomized.<br>Comment: The parameters were inadequately reported.
de Abreu et al., 2024, Portugal (Coimbra)<br>[57]	Human Single-arm study, 15 participants		6-12 months	660	100	-	-	6	1 cm^2	60	26<br>/ 52 weeks	Low	III	The results indicated a statistically significant improvement ($p < 0.001$) in four of the five dimensions of the health-related quality of life questionnaire, namely self-care, usual activities, pain/discomfort, and anxiety/ depression, along with the patients' perceived health level. A total of 13 participants reported suspending or reducing their intake of medications for BMS.

dos Santos Lde et al., 2015, Brazil (São Paulo) [58]	Human Single-arm study, 20 participants		10 weeks	660	40	-	0.4 /point	10	0.04 mm^2 spot size	10 /point	10 / 10 weeks	Serious	III	All volunteers reported reduced burning intensity in all sessions when compared to the previous one and reduction in VAS scores by up to 49% in the last clinical session when compared to the first session.<br>Comment: What is the number of irradiated points? Nothing been reported.
Valenzuela & Lopez-Jornet et al., 2017, Spain (Murcia) [59]	Human Randomised trial, 44 participants (44 completed)	Groups:<br>- laser 133.3 J/cm^2<br>- laser 200 J/cm^2<br>- sham laser	4 weeks	815	1000	-	4<br>6	133<br>200	0.03 cm^2 spot size	4<br>6	4 / 4 weeks	High	II	Overall improvements in VAS scores from baseline to the end of treatment were: Group I 15.7%; Group II 15.6%; Group III placebo 7.3%.<br>Comment: It's not clear whether they mean "4" and "6" OR ".4" and ".6" joules.
Finfter et al.,2024, Israel (Jerusalem) [60]	Human Single-arm study		up to 10 weeks	940	(see full text)	-	-	-	-	90 / each 2.5 cm2 area	varying	Serious	III	Immediate mean VAS score decreased from a starting score of 7.80 ± 1.83 to 2.07 ± 2.55 ($p < 0.001$). The mean weekly VAS score for the week after the final treatment session was higher (5.73 ± 2.80, $p < 0.001$) than the immediate response, but still significantly lower than the starting score ($p = 0.017$). We observed a trend of pain improvement with more treatments, but this was only statistically significant up to the third treatment. Male gender and unilateral pain correlated with better PBM efficacy ($p = 0.017$, 0.022, respectively). Comment: Parameters were inadequately reported.
Marotta et al., 2024, Brazil (São Paulo) [61]	Human Randomised trial, sham-controlled, 30 participants		10 weeks	660	100			6	0.04 cm2 spot size	10 /point	10 / 10 weeks	Serious	III	Results showed no statistically significant difference in reduction of pain intensity between the two groups at all the evaluated timepoints during the course of treatment.<br>However, in both groups, a statistically significant reduction of maximum pain intensity of 50% compared with patient-self reporting before the treatment was noted.
Yang & Huang 2011, Taiwan (Taichung) [62]	Human Single-arm trial, 17 participants (17 completed)		treatment weekly until patient feels no further need -> weekly follow-up for 3 months	800	1500	-	-	105	1 cm^2 spot size<br><br>irradiated: the involved area	70 / 1cm2 area	weekly for varying duration	Serious	III	The results showed an average reduction in pain of 47.6% (ranging from 9.3% to 91.8%)."
Romeo et al., 2010, Italy (L'Aquila) [63]	Human Single-arm trial, 25 participants		4 weeks	650<br>+<br>910	-	-	-	0.53	-	900	8 / 4 weeks	Serious	III	Seventeen patients (68%) had relevant benefits from the treatment with valid reduction of NRS ratings. In 8 cases the differences of NRS rates were not relevant being under the limit of reliability established in study design. In no case there was a worsening of the symptoms.<br>Comment: The parameters were inadequately reported
Mu et al., 2024, China (Guangzhou) [64]	Human Retrospective study, 94 patients		Single session	635	100	0.100	-	2	-	-	1	Serious	III	After standardised LLLT session, 71.3% of patients reported an immediate pain decrease. Compared to pre-LLLT treatment, the VAS immediately post-LLLT was significantly reduced ($P < 0.001$). Mean post-LLLT VAS reduction was 2.2 ± 2.0, equivalent to 39.9% of the initial pain level. Meanwhile, low VAS before treatment, history of smoking or alcohol, xerostomia, and gingival lesions correlated with worse LLLT efficacy. There were no side effects or adverse reactions were noticed by the practitioner or reported by the patients.

Six studies on Idiopathic Trigeminal Neuralgia (TN)-induced NP were deemed eligible for inclusion in the systematic review for the Position Paper Recommendations

First author, Year and Country	Study characteristics	Study Groups	Follow up	PBMT parameters								Overall Risk of bias {RoB: Low, Some concerns, High] [ROBINS: Low, Moderate, Serious]	Level of Evidence [Melnyk B et al., 2011]	Outcome
				λ (nm)	Power (mW)	Irradiance (W/cm²)	Energy (J)/point	Fluence J/cm²	Spot size (cm²)	Irradiation time (s)	Frequency per week/ Rx duration			
Karagözoğlu et al., 2024, Turkey (Gaziantep) [65]	Human Randomized trial, sham-controlled, 45 participants (45 completed)	Groups:<br>- new-generation laser<br>- Nd:YAG laser<br>- Nd:YAG placebo	-	650+ 904<br><br>1064	10+ 22<br><br>250	-<br><br>-	80<br><br>-	-<br><br>8	-<br><br>⌀ = 0.9 mm	300<br><br>60	25 / ~7 weeks<br><br>12 / 4 weeks	High	II	Both LLLTs can be considered alternative treatment modalities for TN, but the GRR laser treatment was more effective than the Nd:YAG laser treatment in reducing pain and improving the quality of life in patients with TN.<br>Comment: Parameters incompletely reported
Al-Azab et al., 2023, Egypt (Cairo) [66]	Human Randomized trial, 126 participants (120 completed), PBM vs electromagnetic therapy	Groups:<br>- medication<br>- medication + LLLT<br>- medication + EMT	2 months	830	15	0.150-0.170	-	-	-	1200	3/week for 2 months	High	II	After treatment, all groups improved significantly, with the laser group outperforming the electromagnetic group by a large margin. For irradiation, LLLT was more effective than EMT in reducing diabetic patients' trigeminal pain, and increasing masseter and temporalis muscles compound action potential amplitude in diabetic patients with TN
Ebrahimi et al., 2018, Iran (Tehran) [67]	Human Randomized trial, double-blind, 30 participants,	Groups:<br>- laser<br>- sham laser	-1	810	200 max.	-	5	6.36	-	25	3 sessions per week/ 3 weeks (total 9 sessions)	Low	II	The severity of pain was lower at the end of treatment in the case compared to the control group so this difference was statistically significant (P=0.003). The severity of pain decreased in both groups over time. Significant difference was noted in this regard between the 2 groups either (P=0.003).<br>Comment: The clinical significance is a bit unclear, since the differences between the groups are not very large.<br>Comment: Parameters were inadequately reported.
Aghamohammadi et al., 2012, Iran (Tabriz) [68]	Human Comparison study, randomized, 42 participants	Groups:<br>- Gass.g.block + PBM<br>- Gass.g.block	6 months	890	-	-	3-10 /point	-	-	-	up to 12	High	II	The severity of pain was significantly lower in group A, from day 7 until the end of the study period (month 6). The number of carbamazepine tablets taken was also significantly lower in group A compared with group B from the initial months until the end of the study period (month 6). The period of a pain-free state was significantly higher in group A than in group B (P<0.00l).<br>Comment: LLLT was very clearly superior in this study.<br>Comment: Parameters were inadequately reported. According to the paper, they used "Mustang 026 Russia, and the probe type was LO3"". The following paper (http://proceedings.spiedigitallibrary.org/proceeding.aspx?articleid=887067) seems to have some data on these Russian lasers, but it seems impossible to retrieve the relevant parameters.<br>Comments: It's unclear how long was the intervention period before the 6-month follow-up... maybe 7 days? This journal isn't indexed in PubMed, and its' ranking is surprisingly poor: "Neurosciences 250/251"
Eckerdal & Bastian et al., 2012, Denmark (Odense) [69]	Human Randomized trial, double-blind, 32 participants	Groups:<br>- laser<br>- sham laser	12 months	832	31	-	2	9.2	-	-	5 weeks Rx duration	Low	II	The results demonstrate that of 16 patients treated with the laser probe, 10 were free from pain after completing treatment and 2 had noticeably less pain, while in 4 there was little or no change. After a one year follow-up, 6 patients were still entirely free from pain. In the group treated with the placebo system, i.e. the non-laser probe, one was free from pain, 4 had less pain, and the remaining 9 patients had little or no recovery. After one year only one patient was still completely free from pain. It is concluded that the present study clearly shows that LLLT treatment, given as described, is an effective method and an excellent supplement to conventional therapies used in the treatment of trigeminal neuralgia.
Walker et al., 1987 (USA) [70]	Human Randomized control trial, 35 participants (18 interventional; 17 Control	Groups<br>Experimental (laser)<br>Control	-	632.5	0.477 Average	47.6	-	-	0.05 at skin	30 sec- week 1<br>45 sec- week 2<br>60 sec week 3-6<br>90 sec- week 7-10	3 session per week/ 10 weeks Rx duration	Low	I	Subjects in the experimental group exhibited a statistically significant reduction in the pain intensity as measured by VAS (p<0.002) and the number of painful episodes. In this study the peripheral and facial trigger points were irradiated but no reporting on the total number of irradiation points on the face and whether the affected nerves in the facial region were irradiated.

Eleven studies on Post-Herpetic Neuralgia (PHN)-induced NP were deemed eligible for inclusion in the systematic review for the Position Paper Recommendations.

First author, Year and Country	Study characteristics	Study Groups	Follow up	PBMT parameters								Overall Risk of bias {RoB: Low, Some concerns, High] [ROBINS: Low, Moderate, Serious]	Level of Evidence [Melnyk B et al., 2011]	Outcome
				λ (nm)	Power (mW)	Irradiance (W/cm²)	Energy (J)/ point	Fluence J/cm²	Spot size (cm²)	Irradiation time (s)	Frequency per week/ Rx duration			
Mukhtar et al., 2020, Pakistan (Islamabad) [71]	Human Single-arm trial, 15 participants	-	8 weeks	650	-	-	-	3.6	-	60	16 / 8 weeks	Serious	III	The final pain score was 0 in 11 patients although their initial pain score was severe in 8 and moderate in 3 patients. In three patients, pain reduced to mild intensity (2-3), and in one, the final pain score was 4 on the visual analogue scale. Overall, low-level laser therapy (LLLT) proved itself an excellent therapeutic modality for the relief of pain in post-herpetic neuralgia patients, which may replace pain management medicines in future. Comment: Parameters were inadequately reported
Park et al., 2013, Korea (Seoul) [72]	Human Randomized trial, 28 participants	LED phototherapy Groups: (A) conventional + famcyclovir (B) conventional + famcyclovir + PBM	10-day treatment / 14-day study	830	-	0.055	-	-	-	600	4 / 10 days	High	II	The mean time required for wound healing was 13.14±2.34 days in group B and 15.92±2.55 days in group A (p=0.006). From day 4, the mean VAS score showed a greater improvement in group B, compared with group A. A marginal but not statistically significant difference in the VAS scores was observed between the two groups (p=0.095). Comment: Anatomical site and application mode, and some treatment parameters, were inadequately reported.
Mann et al., 1999 (India) [73]	Human Single-arm trial, 50 participants		Weekly for 8 weeks	-	-	-	-	-	-	5 mins and 65 secs	1/15 days	Serious	III	Patients started responding to the therapy after a n average of 3.28 laser applications and VAS steadily decreased as the therapy progressed. After completion of therapy, 34 (86%) out of 50 cases showed excellent relief (76-100%) and remaining 7(14%) cases showed partial relief. Combi laser therapy gave excellent results in cases of PHN but it was comparatively less effective i.e. it gave partial pain relief in cases with prolonged duration (more than 2 years), in cases of ophthalmic herpes zoster and in cases with keloid and scarring formation.
Toshikazu et al., 1997, Japan (Hokkaido) [74]	Human Randomized trial, crossover, double-blind, 8 participants	Dose response Crossover conditions: laser 60 nW; laser 180 mW, vs sham	-	830	60 (MLD-1002); 150 (MLD-1003	-	-	85.9<br><br>214.8	4 mm	180 s for all the groups	X2 (1x for 60 mW and 1x for 180 mW (crossover study)	Low	II	Regional skin temperature increased following both 150 mW and 60mW laser irradiation, whereas no changes were obtained by placebo treatment. VAS decreased following both 150 mW and 60 mW laser treatments, but no changes in VAS were obtained by placebo treatment. These changes in the temperature and VAS were further dependent on the energy density, i.e the dose. Results demonstrate that laser irradiation near the stellate ganglion produces effects similar to stellate ganglion block. Our results clearly indicate that they are not placebo effects but true effects of laser irradiation. Comment: Parameters were inadequately reported.
Hiromichi 1995, Japan (Tokyo) [75]	Human Comparison study, non-randomized, 17 participants	Dose response PBM (60mW) vs PBM (150mW)	Treatment until pain score adequately low (~ 1-2 months)	830	60<br>60<br>150<br>150	-	10.8<br>72<br>27<br>180	-	-	180<br>1200<br>180<br>1200	1-5/week for outpatients 5-6/week for inpatients approx. 9-13 on average	Serious	III	Our results in this preliminary study suggest that 150 mW LLLT was a much more effective therapy than 60 mW LLLT for the treatment of PHN within the period tested. Further controlled and double blinded studies in larger populations will be required to corroborate these preliminary findings. Comment: Irradiation duration (3-20min) depended on size of affected area. Comment: Study methodology very irregular. The patients received very varying doses etc.
Iijima et al., 1991, Japan (Chiba) [76]	Human Single-arm trial, 18 participants	-	8.1 ± 3.3 months	633	8.5	-	-	-	⌀ = 0.1 mm	1440 (3min / point) (8 points)	2-3 per week up to 50	Serious	III	3.6 after 50 treatments, and the degree of pain relief was reduced to 44.6% and correlated with the number of treatments. The total numbers of words and the total scores of the m-MPQ decreased as the number of treatments increased.
Kemmostsu et al., 1991, Japan (Sapporo) [77]	1.Human Single-arm trial, 63 participants 2. Human (suffering for more than 1 year), Non-randomized study, crossover, double-blind, 12 participants	-	1.avg duration of 36 sessions 2. Single session (5min treatment -> 5min washout -> 5min treatment)	830 (MLD-2001 model)	60	~ 1.2 - 3	-	-	-	10 per point	2-3 / outpatients 4-6 / inpatients per week total 36 on average	Low	III	The long-term effect at the end of LLLT (the average number of treatments 36 ± 12) resulted in no pain (PS: 0) in 12 patients and slight pain (PS: 1-4) in 46 patients, No complications attributable to LLLT occurred. Although a placebo effect was observed, decreases in pain scores and increases of the body surface temperature by LLLT were significantly greater than those that occurred with the placebo treatment. The results indicate that LLLT is a useful modality for pain attenuation in PHN patients and because LLLT is a non-invasive, painless and safe method of therapy, it is well acceptable by patients. Comment: Parameters were inadequately reported
Lloyd et al 1991, Canada (Wheatley) [78]	Human Single-arm trial, 39 participants	-	Treatment course --> 1-year follow-up	904	6	-	-	~4	-	1200	5 (at least 4h between irradiations)	Serious	III	The reported level of PHN pain decreased significantly after treatment ($p < 0.05$). Comment: Parameters inadequately reported. The course of treatment is somewhat unclearly described as well.
Hong et al., 1990, Korea (Incheon) [79]	Human Single-arm trial, 20 participants	Treatment-resistant patients	~1 year	830	60	-	-	-	-	300-600	1-44 sessions twice weekly average 21	Serious	III	Twelve of the 20 patients responded to the LLLT, 60%. Further studies are needed. with emphasis on methodology. as these figures are not as high as those from other workers in this field, The success rate was high enough however to make the authors consider a larger programme in the future, including double-blind and crossover components. The GaAlAs diode laser in contact LLLT certainly provides a new non-invasive and easily applied therapy

														for PHN.<br>Comment: Parameters inadequately reported.
Iijima et al., 1989, Japan (Chiba) [80]	Human Single-arm trial, 36 participants	-	for 20 sessions (7-10 weeks)	633	8	-	-	-	⌀ = 0.1 mm	180/point (6 or 8 points) = total 1010 sec on avg	20 (2-3 per week)	Serious	III	VAS decreased from 6.2 before irradiation therapy to 3.6 after 50 treatments, and the degree of pain relief was reduced to 44.6% and correlated with the number of treatments. The total numbers of words and the total scores of the m-MPQ decreased as the number of treatments increased.
Moore et al., 1988, UK (Oldham) [81]	Human Randomized trial, double-blind, crossover, 20 participants with mean of two and half years of PHN	Treatment-resistant patients. Groups: A: laser -> sham B: sham -> laser	1 month (2 weeks per condition)	830	60	3	-	45	0.05	15/point	4 active, 4 sham twice weekly	Low	II	Measurements of pain intensity and distribution were noted over a period of eight treatments in two groups of patients each of which received four consecutive laser treatments. The results demonstrate a significant reduction in both PHN pain intensity and distribution following a course of LLLT.
