# Supplementary material for: Photobiomodulation Therapy in the Management of Orofacial Neuropathic Pain—WALT Position Paper 2026
Source: J Clin Med. 2026 Feb 6;15(3):1304. doi: 10.3390/jcm15031304 (PMC12898000; doi:10.3390/jcm15031304)
Supplement: Supplementary file 1 [file jcm-15-01304-s001.zip › Supplementary File S7-Figures S1-S10.pdf]

Study	Condition	AMSTAR Criteria Questions (green= non-critical, red= critical)																Score
		1	2	3	4	5	6	7	8	9	10	11	12	13	14	15	16	
Ibarra et al	TN	✓	✓	X	✓	✓	X	✓	✓	✓	X	X	X	✓	X	X	✓	Critically Low
Hanna et al	BMS	✓	✓	✓	✓	✓	✓	✓	✓	✓	X	✓	✓	✓	✓	✓	✓	High
Okuhara et al	BMS	✓	✓	✓	✓	✓	✓	✓	✓	✓	X	✓	✓	✓	✓	-	✓	Moderate
de Lima et al	BMS	X	✓	X	✓	✓	✓	✓	✓	✓	X	X	X	✓	✓	X	X	Critically Low
Camolesi et al	BMS	✓	✓	✓	✓	✓	✓	✓	✓	✓	X	✓	✓	✓	X	X	X	Moderate
Zhang et al	BMS	✓	✓	✓	✓	✓	✓	✓	✓	✓	X	✓	✓	✓	✓	-	X	Moderate
Sun et al	BMS	✓	X	✓	●	X	X	X	✓	✓	✓	✓	✓	✓	✓	X	✓	Critically Low
Pedro et al	ON/GN/PTTN/BMS/TN	✓	X	X	✓	✓	✓	✓	✓	✓	X	X	X	✓	✓	X	✓	Critically Low

Figure S1. AMSTAR 2 assessment of the methodological quality of the included systematic reviews and meta-analyses. Each review was evaluated using the AMSTAR 2 tool, and the level of criticality was determined based on the presence of critical and non-critical weaknesses. The overall confidence rating in the results of each review is categorised as high, moderate, low, or critically low, in accordance with AMSTAR 2 guidelines. Abbreviations: tick is "yes" answer to the questions in the Amstar guidance, cross is a "no" and "dot is a "partial yes".

Study ID	Experimental	Comparator	Outcome	D1	D2	D3	D4	D5	Overall
Lu 2025	PBM	OCT	Pain	+	!	+	+	+	!
Ge 2025	PBM	PBM	Pain	+	!	+	+	+	!
Martinez 2024	PBM	Clonazepam	Pain	+	+	+	!	+	!
Medeiros 2023	PBM	TENS	Pain	+	-	+	-	+	-
Loncar-Brzak 2022	PBM	Vit B/Probiotics	QoL	+	-	+	-	+	-
Scardina 2020	PBM	Placebo	Pain	!	-	+	+	+	-
Skrinjar 2020	PBM	Placebo	Pain	-	-	-	-	!	-
De Pedro 2020	PBM	Placebo	Pain	+	+	+	+	+	+
Bardellini 2019	PBM	Placebo	Pain	+	+	+	+	+	+
Spanemberg 2019	PBM	Placebo	Pain	-	-	+	-	+	-
Sikora 2018	PBM	Placebo	Pain	!	-	+	-	+	-
Barbosa 2018	PBM	ALA	Pain	+	!	+	+	+	!
Valenzuela 2017	PBM	Placebo	Pain	+	!	+	-	+	-
Sugaya 2016	PBM	Placebo	Pain	+	+	+	+	+	+
Arduino 2016	PBM	Medication	Pain	+	+	+	+	+	+
Arbabi-Kalati 2015	PBM	Placebo	Pain	+	!	+	+	+	!
Spanemberg 2015	PBM	Placebo	Pain	-	-	+	-	+	-
Pezelj- Ribaric 2013	PBM	Placebo	Pain	-	-	+	-	+	-

Figure S2. Tabular representation of the RoB-2 assessment of included RCTs on utilisation of PBM in the treatment of burning mouth syndrome

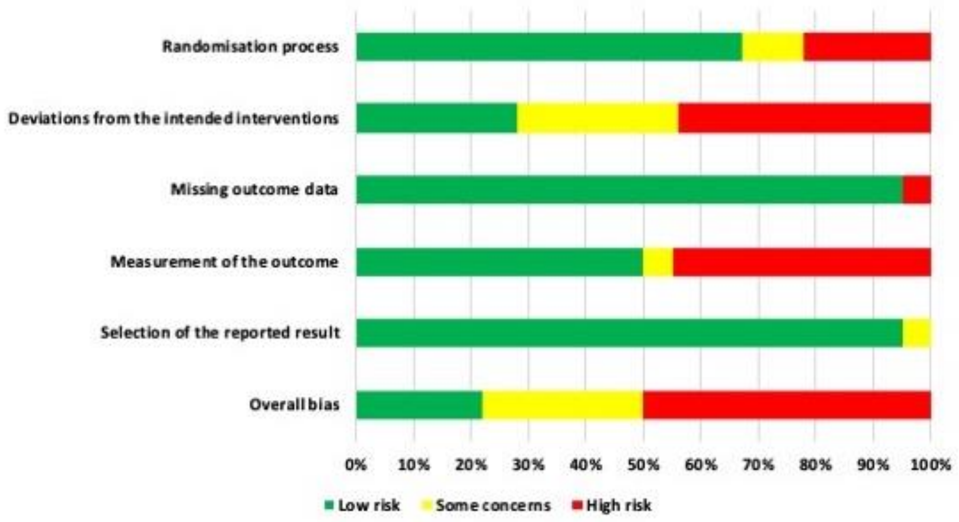

Figure S3. Graphical representation of the RoB-2 assessment of included RCTs on utilisation of PBM in the treatment of burning mouth syndrome

Author	Domains							Overall risk of bias
	Pre-intervention		Intervention	Post-intervention				
	Bias due to confounding	Bias in selection of participants for the study	Bias in classifying interventions	Bias due to deviations from intended interventions	Bias due to missing data	Bias to measuring outcomes	Bias in selecting reported results	
De Abreu 2024	Low	Low	Low	Low	Low	Low	Low	Low
Finfter 2024	Low	Low	Low	Low	Low	Serious	Low	Serious
Marrotta 2024	Low	Low	Low	Low	Low	Serious	Low	Serious
Hanna 2022	Low	Low	Low	Low	Low	Low	Low	Low
Santos 2015	Low	Low	Low	Low	Low	Serious	Low	Serious
Yang & Huang 2011	Low	Low	Low	Low	Low	Serious	Low	Serious
Romeo 2010	Low	Low	Low	Low	Low	Serious	Low	Serious

Figure S4. Tabular representation of the ROBINS-I assessment of included NRCTs on utilisation of PBM in the treatment of burning mouth syndrome.

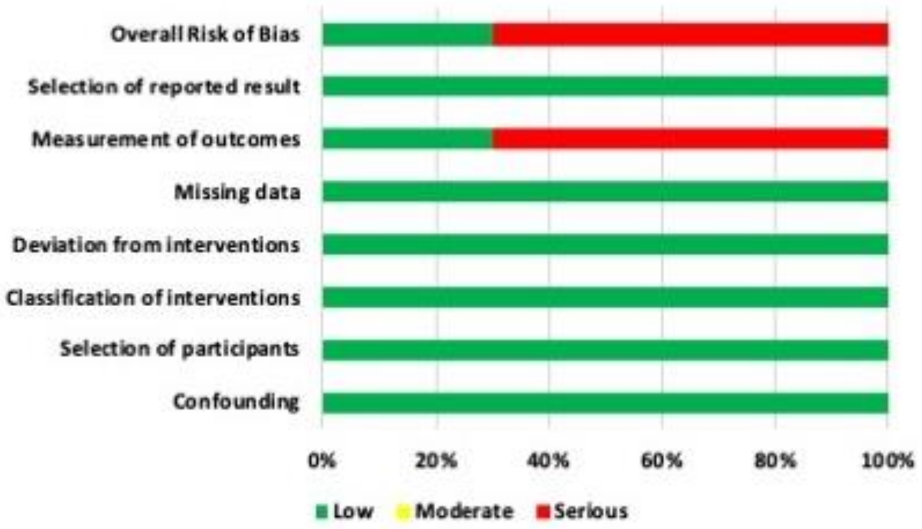

Figure S5. Graphical representation of the ROBINS-I assessment of included NRCTs on utilisation of PBM in the treatment of burning mouth syndrome

Study ID	Experimental	Comparator	Outcome	D1	D2	D3	D4	D5	Overall
Karagözog̃lu et al., 2024	LLLT	Placebo	Pain, QoA	!	+	+	-	+	-
Al-Azab et al., 2023	LLLT	EMT	Pain	+	-	+	-	+	-
Ebrahimi et al., 2018	LLLT	Pharmaceutical therapy	Pain	+	+	+	+	+	+
Aghamohammadi et al., 2012	LLLT+Trigeminal ganglion block	Trigeminal ganglion block	Pain	+	+	+	-	+	-
Eckerdal et al., 1996	LLLT	Placebo	Pain	+	+	+	+	+	+
Walker et al., 1987 (USA)	LLLT	Placebo	Pain	+	+	+	+	+	+

Figure S6. Tabular representation of the RoB-2 assessment of included RCTs on utilisation of PBM in the treatment of trigeminal neuralgia

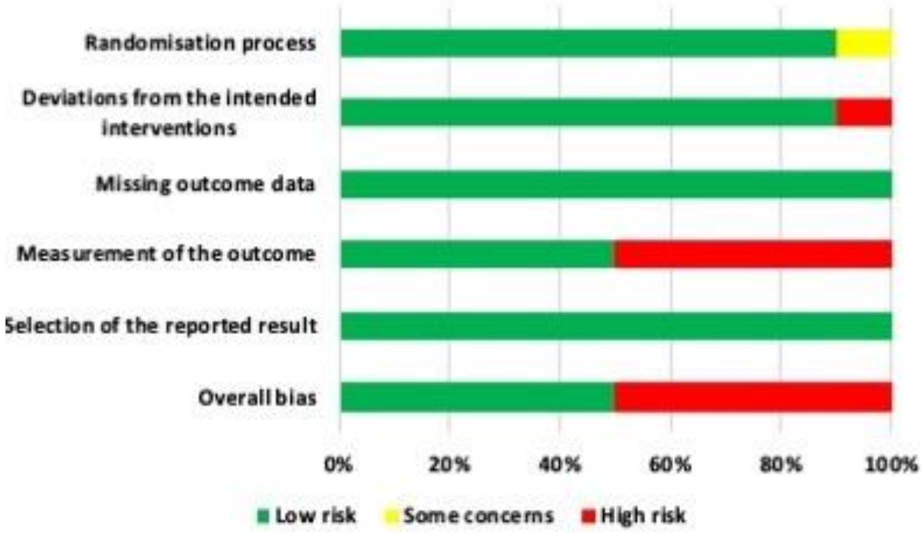

Figure S7. Graphical representation of the RoB-2 assessment of included RCTs on utilisation of PBM in the treatment of trigeminal neuralgia

Author	Domains							Overall risk of bias
	Pre-intervention		Intervention	Post-intervention				
	Bias due to confounding	Bias in selection of participants for the study	Bias in classifying interventions	Bias due to deviations from intended interventions	Bias due to missing data	Bias to measuring outcomes	Bias in selecting reported results	
Mukhtar et al., 2020	Low	Low	Low	Low	Low	Serious	Low	Serious
Mann et al., 1999	Low	Low	Low	Low	Low	Serious	Low	Serious
Hiromichi et al., 1995	Low	Low	Low	Low	Low	Serious	Low	Serious
Iijima et al., 1991	Low	Low	Low	Low	Low	Serious	Low	Serious
Kemmostsu et al., 1991	Low	Low	Low	Low	Low	Low	Low	Low
Llyod et al., 1991	Low	Low	Low	Low	Low	Serious	Low	Serious
Hong et al.,1990	Low	Low	Low	Low	Low	Serious	Low	Serious
Iijima et al.,1989	Low	Low	Low	Low	Low	Serious	Low	Serious

Figure S8. Tabular representation of the ROBINS-I assessment of included NRCTs on utilisation of PBM in the treatment of post-herpetic neuralgia.

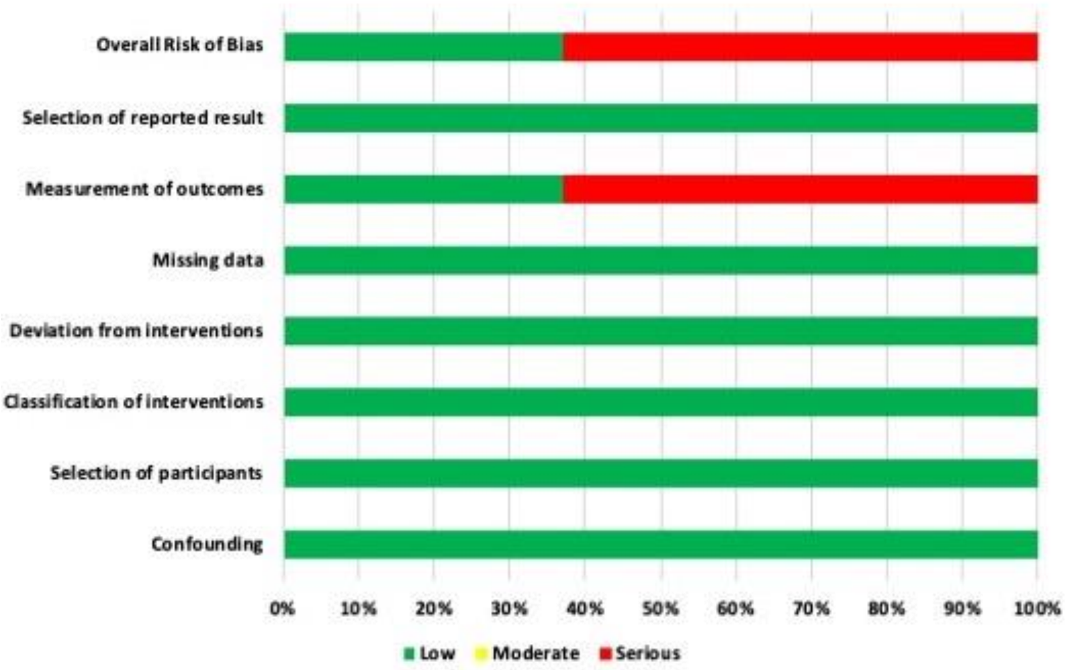

Figure S9. Graphical representation of the ROBINS-I assessment of included NRCTs on utilisation of PBM in the treatment of post-herpetic neuralgia.

Study ID	Experimental	Comparator	Outcome	D1	D2	D3	D4	D5	Overall
Park et al., 2013	LLLT	Oral Famyclovir	Pain	!	+	+	-	+	-
Toshikazu et al., 1997	LLLT	Sham Laser	Pain	+	+	+	+	+	+
Moore et al.,1988	LLLT	Sham Laser	Pain	+	+	+	+	+	+

Figure S10. Tabular representation of the RoB-2 assessment of included RCT on utilisation of PBM in the treatment of post-herpetic neuralgia
